# Observation weights unlock bulk RNA-seq tools for zero inflation and single-cell applications

**DOI:** 10.1186/s13059-018-1406-4

**Published:** 2018-02-26

**Authors:** Koen Van den Berge, Fanny Perraudeau, Charlotte Soneson, Michael I. Love, Davide Risso, Jean-Philippe Vert, Mark D. Robinson, Sandrine Dudoit, Lieven Clement

**Affiliations:** 1 Department of Applied Mathematics, Computer Science and Statistics, Ghent University, Krijgslaan 281, S9, Ghent, 9000 Belgium; 2 Bioinformatics Institute Ghent, Ghent University, Ghent, 9000 Belgium; 3 Division of Biostatistics, School of Public Health, University of California, Berkeley, USA; 4 Institute of Molecular Life Sciences, University of Zurich, Winterthurerstrasse 190, Zurich, 8057 Switzerland; 5 SIB Swiss Institute of Bioinformatics, University of Zurich, Zurich, 8057 Switzerland; 6 Department of Biostatistics and Genetics, The University of North Carolina at Chapel Hill, Chapel Hill, NC USA; 7 Division of Biostatistics and Epidemiology, Department of Healthcare Policy and Research, Weill Cornell Medicine, New York, USA; 8 MINES ParisTech, PSL Research University, CBIO-Centre for Computational Biology, Paris, France; 9 Institut Curie, Paris, France; 10 INSERM U900, Paris, France; 11 Ecole Normale Supérieure, Department of Mathematics and Applications, Paris, France; 12 Department of Statistics, University of California, Berkeley, USA

**Keywords:** Single-cell RNA sequencing, Differential expression, Zero-inflated negative binomial, Weights

## Abstract

Dropout events in single-cell RNA sequencing (scRNA-seq) cause many transcripts to go undetected and induce an excess of zero read counts, leading to power issues in differential expression (DE) analysis. This has triggered the development of bespoke scRNA-seq DE methods to cope with zero inflation. Recent evaluations, however, have shown that dedicated scRNA-seq tools provide no advantage compared to traditional bulk RNA-seq tools. We introduce a weighting strategy, based on a zero-inflated negative binomial model, that identifies excess zero counts and generates gene- and cell-specific weights to unlock bulk RNA-seq DE pipelines for zero-inflated data, boosting performance for scRNA-seq.

**Electronic supplementary material:**

The online version of this article (10.1186/s13059-018-1406-4) contains supplementary material, which is available to authorized users.

## Background

Transcriptomics has become one of the standard tools in modern biology for unraveling the molecular basis of biological processes and diseases. One of the most common applications of transcriptome profiling is the discovery of *differentially expressed* (DE) genes, which exhibit changes in expression levels across conditions [1–3]. Over the last decade, transcriptome sequencing (RNA-seq) has become the standard technology for transcriptome profiling, enabling researchers to study average gene expression over bulks of thousands of cells [4, 5]. The advent of single-cell RNA-seq (scRNA-seq) enables high-throughput transcriptome profiling at the resolution of single cells and allows, among other things, research on cell developmental trajectories, cell-to-cell heterogeneity, and the discovery of novel cell types [6–11].

In scRNA-seq, individual cells are first captured, their RNA is then reverse-transcribed into cDNA, which is greatly amplified from the minute amount of starting material, and the resulting library is finally sequenced [12]. Transcript abundances are typically estimated by counts that represent the number of sequencing reads mapping to an exon, transcript, or gene. Many scRNA-seq protocols have been published for such core steps [13–18], but despite these advances, scRNA-seq data remain inherently noisy. *Dropout* events cause many transcripts to go undetected for technical reasons, such as inefficient cDNA polymerization, amplification bias, or low sequencing depth, leading to an excess of zero read counts compared to bulk RNA-seq data [18, 19]. In addition, excess zeros can also occur for biological reasons, such as transcriptional bursting [20]. There are, therefore, two types of zeros in scRNA-seq data: *biological zeros*, when a gene is simply not expressed in the cell, and *technical zeros* (i.e., dropouts), when a gene is expressed in the cell but not detected. *Zero inflation*, i.e., excess zeros compared to standard count distributions (e.g., negative binomial) used in bulk RNA-seq, occurs for both biological and technical reasons and disentangling the two sources is not trivial. In addition, scRNA-seq counts are inherently more variable than bulk RNA-seq counts because the transcriptional signal is not averaged across thousands of individual cells (Additional file 1: Figure S1), making cell-to-cell heterogeneity, cell-type mixtures, and stochastic expression bursts important contributors to between-sample variability [7, 21].

Typical scRNA-seq data analysis workflows often involve identifying cell types in silico using tailored clustering algorithms [22, 23] or ordering cells along developmental trajectories, where cell types are defined as terminal states of the developmental process [6, 24–26]. A natural subsequent step is the discovery of marker genes for the defined cell types by assessing differential gene expression between these groups. Another common setting is the identification of marker genes for a priori known cell types. DE analysis between homogeneous cell populations, as in the aforementioned scRNA-seq applications, is the use case for our method.

Popular bulk RNA-seq DE tools, such as those implemented in the Bioconductor R packages EDGER [2] and DESEQ2 [1], assume a negative binomial (NB) count distribution across biological replicates, while limma-voom [3] uses linear models for log-transformed counts and observation-level weights to account for the mean–variance relationship of the transformed count data. Such tools can also be applied for scRNA-seq DE analysis [27]. However, dropouts, transcriptional bursting, and high variability in scRNA-seq data raise concerns about their validity. This has triggered the development of novel dedicated tools, which typically introduce an additional model component to account for the excess of zeros through, for example, zero-inflated (SCDE, Kharchenko et al. [28]) or hurdle (MAST, Finak et al. [19]) models. However, Jaakkola et al. [29] and Soneson and Robinson [30] have recently shown that these bespoke tools do not provide systematic benefits over standard bulk RNA-seq tools in scRNA-seq applications.

**Fig. 1:**
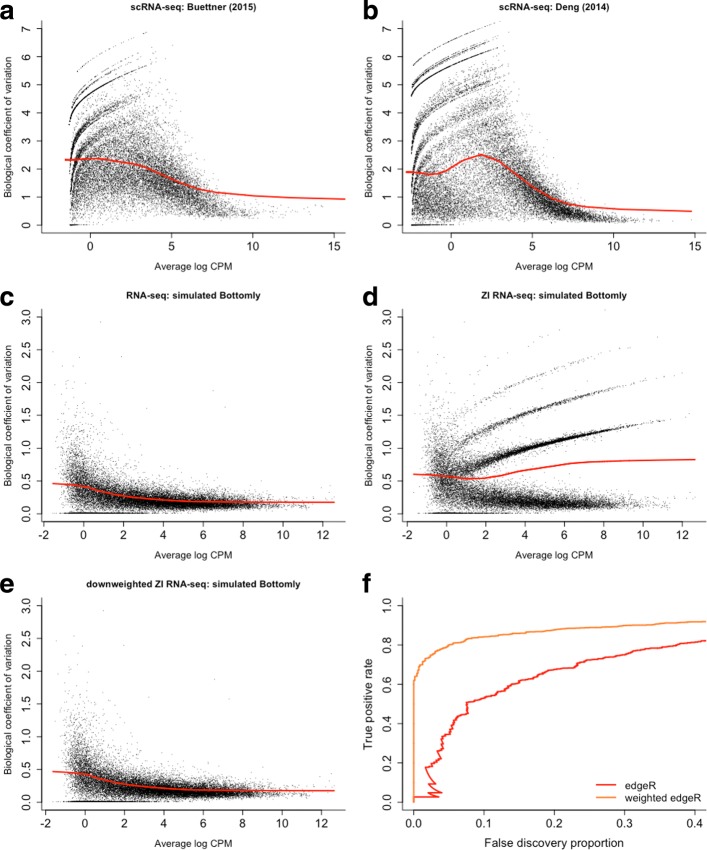
Zero inflation results in overestimated dispersion and jeopardizes power to discover differentially expressed genes. **a–e** Scatterplots of the estimated biological coefficient of variation (BCV, defined as the square root of the negative binomial dispersion parameter *ϕ*) against average log counts per million (CPM) computed using EDGER. **a** BCV plot for the real Buettner et al. [7] scRNA-seq dataset subsampled to *n*=10 cells. **b** BCV plot for the real Deng et al. [66] scRNA-seq dataset subsampled to *n*=10 cells. Both panels (**a**) and (**b**) show striped patterns in the BCV plot, which significantly distort the mean–variance relationship, as represented by the red curve. **c** BCV plot for a simulated bulk RNA-seq dataset (*n*=10), obtained from the Bottomly et al. [67] dataset using the simulation framework of Zhou et al. [57]. Dispersion estimates generally decrease smoothly as gene expression increases. **d** BCV plot for a simulated zero-inflated bulk RNA-seq dataset, obtained by randomly introducing 5% excess zero counts in the dataset from (**c**). Zero inflation leads to overestimated dispersion for the genes with excess zeros, resulting in striped patterns, as observed also for the real scRNA-seq data in panels (**a**) and (**b**). **e** BCV plot for simulated zero-inflated bulk RNA-seq dataset from (**d**), where excess zeros are downweighted in dispersion estimation (i.e., weights of 0 for excess zeros and 1 otherwise). Downweighting recovers the original mean–variance trend. **f** True positive rate vs. false discovery proportion for the simulated zero-inflated dataset of (**d**). The performance of EDGER (red curve) deteriorates in a zero-inflated setting due to overestimation of the dispersion parameter. However, assigning the excess zeros a weight of zero in the dispersion estimation and model fitting result in a dramatic performance boost (orange curve). Hence, downweighting excess zero counts is the key to unlocking bulk RNA-seq tools for zero inflation. BCV biological coefficient of variation, CPM counts per million, ZI zero inflated

We argue that standard bulk RNA-seq tools, however, still suffer in performance due to zero inflation with respect to the NB distribution. We illustrate this using biological coefficient of variation (BCV) plots [31], which represent the mean–variance relationship of the counts. Note that the BCV plots of scRNA-seq data exhibit striped patterns (Fig. 1a,b and Additional file 1: Figure S2 for scRNA-seq datasets subsampled to ten cells), which are indicative of genes with few positive counts (Additional file 1: Figure S3) and very high dispersion estimates. Randomly adding zeros to bulk RNA-seq data, likewise consisting of ten samples, also results in similar striped patterns (Fig. 1c,d). NB models, as implemented in DESEQ2 and EDGER, will, thus, accommodate excess zeros by overestimating the dispersion parameter, which jeopardizes the power to infer DE. However, by correctly identifying the excess zeros and downweighting them in the dispersion estimation and model fitting, one can reconstruct the original mean–variance relationship (Fig. 1e), thus recovering the power to detect DE (Fig. 1f). Hence, identifying and downweighting excess zeros are the key to unlocking bulk RNA-seq tools for scRNA-seq DE analysis. Note that methods based on a zero-inflated negative binomial (ZINB) model naturally implement such an approach. Excess zeros are attributed weights through the zero-inflation probability and inference can focus on the mean of the NB count component.

We, therefore, propose a weighting strategy based on ZINB models to unlock bulk RNA-seq tools for scRNA-seq DE analysis. In this manuscript, we build on the ZINB-based wanted variation extraction (ZINB-WaVE) method of Risso et al. [23], designed specifically for scRNA-seq data. ZINB-WaVE efficiently identifies excess zeros and provides gene- and cell-specific weights to unlock bulk RNA-seq pipelines for zero-inflated data. As most bulk RNA-seq DE methods are based on generalized linear models (GLMs), which readily accommodate observation-level weights, our approach seamlessly integrates with standard pipelines (e.g., EDGER, DESEQ2, and LIMMA). Our method is shown to outperform competing methods on simulated bulk and single-cell RNA-seq datasets. We also illustrate our method on two publicly available real datasets. As detailed in “Software implementation,” our approach is implemented in open-source Bioconductor R packages and the code for reproducing the analyses presented in this manuscript is provided in a GitHub repository.

## Results

### ZINB-WaVE extends bulk RNA-seq tools to handle zero-inflated data

We argue that standard bulk RNA-seq methods for inferring differential gene expression suffer from zero inflation with respect to the assumed NB distribution when applied to scRNA-seq data. We propose instead modeling scRNA-seq data using a zero-inflated model and perform inference on the count component of the model, which is equivalent to standard NB regression where excess zeros are downweighted based on posterior probabilities (weights) inferred from a ZINB model. Such weights play a central role in many estimation approaches for ZINB models (e.g., [32]). In this contribution, we show that the weights can effectively unlock bulk RNA-seq methods for zero-inflated data, allowing us, in particular, to borrow strength across genes to estimate dispersion parameters. Here, we use weights derived from the ZINB-WaVE method of Risso et al. [23], which is a general and flexible framework for the extraction of a low-dimensional signal from scRNA-seq read counts, accounting for zero inflation (i.e., dropouts and bursting), over-dispersion, and the discrete nature of the data. Note that although we focus on ZINB-WaVE weights, our weighted DE approach is generic and researchers can choose to adopt their own weights.

A ZINB distribution is a two-component mixture between a point mass at zero and a NB distribution. Specifically, the density function for the ZINB-WaVE model is

1 $$ {}{{\begin{aligned} f_{\text{ZINB}}\left(y_{ij};\mu_{ij}, \theta_{j}, \pi_{ij}\right) = \pi_{ij}\delta_{0}\left(y_{ij}\right) + \left(1-\pi_{ij}\right)f_{\text{NB}}\left(y_{ij}; \mu_{ij}, \theta_{j}\right), \end{aligned}}}  $$

where *y*_*ij*_ denotes the read count for cell *i* and gene *j*, *π*_*ij*_ the mixture probability for zero inflation, *f*_NB_(·;*μ*_*ij*_,*θ*_*j*_) the NB probability mass function with mean *μ*_*ij*_ and dispersion *θ*_*j*_, and *δ*_0_ the Dirac delta function (see Eqs. 3 and 4).

The ZINB-WaVE parameterization of the NB mean *μ* and zero-inflation probability *π* in Eq. 4 allows us to adjust for both known (e.g., treatment, batch, and quality control measures) and unknown (RUV (remove unwanted variation)) [33, 34] cell-level covariates, i.e., supervised and unsupervised normalization, respectively. It also allows us to adjust for known gene-level covariates (e.g., length and GC content). The ZINB-WaVE model and its associated penalized maximum likelihood estimation procedure are described more fully in “Methods” and in Risso et al. [23].

From the ZINB-WaVE density of Eq. 1, one can readily derive the posterior probability that a count *y*_*ij*_ was generated from the NB count component:

2 $$ w_{ij} = \frac{\left(1 - \pi_{ij}\right) f_{\text{NB}}\left(y_{ij}; \mu_{ij}, \theta_{j}\right) }{f_{\text{ZINB}}\left(y_{ij};\mu_{ij}, \theta_{j}, \pi_{ij}\right)}.  $$

We propose using these probabilities as weights in bulk RNA-seq DE analysis methods, such as those implemented in the Bioconductor R packages EDGER, DESEQ2, and LIMMA (limma-voom method with the voom function). All of these methods are based on the methodology of GLMs, which readily accommodates inference based on observation-level weights. Note that although the ZINB-WaVE weights are gene- and cell-specific, the GLMs are fitted gene by gene. Hence, for a given gene, the cell-specific weights are used as observation-specific weights in the GLMs. The implementation of the weighting strategy for EDGER, DESEQ2, and limma-voom is described in greater detail in “Methods.”

### Impact of zero inflation on the mean–variance relationship

**Fig. 2:**
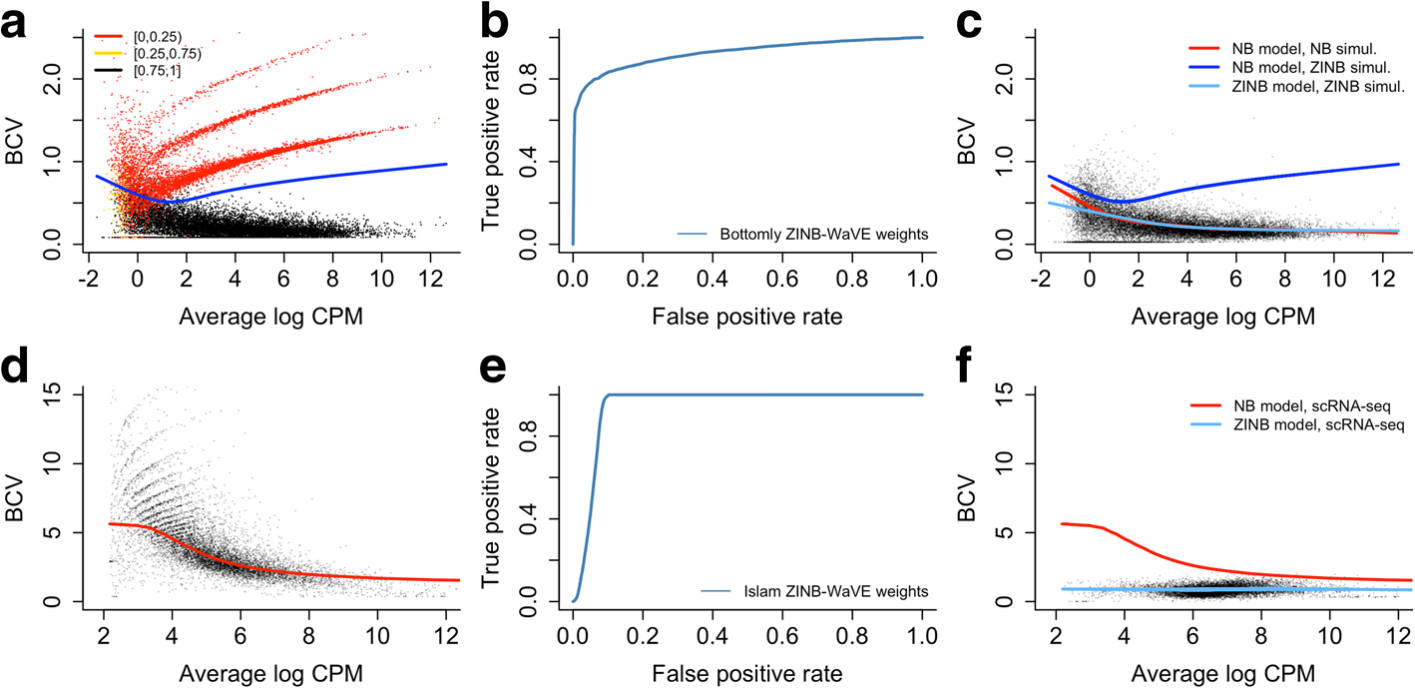
Impact of zero inflation on mean–variance relationship for simulated bulk RNA-seq and Islam scRNA-seq datasets. Zero inflation distorts the mean–variance trend in (single-cell) RNA-seq data, but is correctly identified by the ZINB-WaVE method. The top panels represent simulated data based on the Bottomly et al. [67] bulk RNA-seq dataset (as in Fig. 1), for a two-group comparison with five samples in each group, where 5% of the counts were randomly replaced by zeros. The bottom panels represent the scRNA-seq dataset [35] from Islam et al. [16]. **a** The BCV plot shows that randomly replacing 5% of the read counts with zeros induces zero inflation and distorts the mean–variance trend through overestimating the dispersion parameters. Points are color-coded according to the average ZINB-WaVE posterior probability for all zeros for a given gene and the blue line represents the mean–variance trend estimated with EDGER. **b** Receiver operating characteristic (ROC) curve for identifying excess zeros by the ZINB-WaVE method. A very good classification precision is obtained. **c** Downweighting excess zeros using the ZINB-WaVE posterior probabilities recovers the original mean–variance trend (as indicated with the red line) and inference on the NB count component will now no longer be biased because of zero inflation. The light blue line represents the estimated mean–variance trend for ZINB-WaVE-weighted EDGER. The blue line is the trend estimated by unweighted EDGER on zero-inflated data as in panel (**a**). **d** The BCV plot for the Islam et al. [16] dataset illustrates the higher variability of scRNA-seq data compared to bulk RNA-seq data. Note the difference in *y*-axis scales between (**a**) and (**d**). As in (**a**), zero inflation induces striped patterns leading to an overestimation of the NB dispersion parameter. **e** ROC curve for the identification of excess zeros by the ZINB-WaVE method for scRNA-seq data simulated from the Islam dataset using the simulation framework described in “Methods.” A good classification precision is obtained, but note the difference with bulk RNA-seq data. The noisier scRNA-seq dataset makes identification of excess zeros harder. **f** Using the ZINB-WaVE posterior probabilities as observation weights results in lower estimates of the dispersion parameter, unlocking powerful differential expression analysis with standard bulk RNA-seq differential expression methods. Note that since many zeros are identified as excess, the scale of the BCV plot is now similar to that of a standard bulk RNA-seq dataset. The red line is the mean–variance trend for unweighted EDGER, as in panel (**d**), and the light blue line is the mean–variance trend for ZINB-WaVE-weighted EDGER. A similar pattern is observed for the simulated Islam dataset (Additional file 1: Figure S26). BCV biological coefficient of variation, CPM counts per million, NB negative binomial, ROC, receiver operating characteristic, ZINB zero-inflated negative binomial

We have already noted that adding zeros to bulk RNA-seq data results in an overestimation of the dispersion parameter. This leads to striped patterns in the BCV plot (Fig. 2a), which are indicative of genes with many zeros (Additional file 1: Figure S3) and very high dispersion estimates. Our ZINB-WaVE method, however, identifies many of the introduced excess zeros as such (Fig. 2a,b), by classifying them in the zero-inflation component of the ZINB mixture distribution. Using our posterior probabilities as observation-level weights in EDGER recovers the original BCV plot and mean–variance trend (Fig. 2c), illustrating the ability of our method to account for zero inflation. Hence, observation weights provide the key to unlocking standard bulk RNA-seq tools for zero-inflated data.

The BCV plot for the Islam et al. [16] scRNA-seq dataset (Fig. 2d) [35] shows similar striped patterns as for zero-inflated bulk RNA-seq data. Such patterns are observed in many single-cell datasets (Additional file 1: Figure S2). ZINB-WaVE identifies many zeros to be excess for the Islam dataset. It also provides good classification power for excess zeros for data simulated from the Islam dataset (Fig. 2e). Incorporating the ZINB-WaVE weights in an EDGER analysis removes the striped patterns and yields a BCV plot that is similar to that for bulk RNA-seq data (Fig. 2f), suggesting that zero inflation was indeed present and accounted for.

### High power and false positive control on simulated (sc)RNA-seq data

We provide a scRNA-seq data simulation paradigm that retains gene-specific characteristics as well as global associations across all genes (see “Methods” for details). More specifically, we first estimate dataset-specific associations between zero abundance, sequencing depth, and average log counts per million (CPM), and then explicitly account for these associations in our simulation model (Additional file 1: Figures S4, S5).

The scRNA-seq simulation study is based on three datasets: the Islam et al. [16] dataset [35], comparing 48 embryonic stem cells to 44 embryonic fibroblasts in mouse; a subset of the Trapnell et al. [36] dataset, comparing differentiating human myoblasts at the 48 h (85 cells) and 72 h (64 cells) timepoints; and a 10x Genomics peripheral blood mononuclear cell (PBMC) dataset (see “Real datasets” in “Methods” for details). The datasets differ in throughput, sequencing depth, and extent of zero inflation. For example, Additional file 1: Figure S6 shows a higher proportion of excess zeros in the Islam dataset compared to the Trapnell dataset, an observation further supported because the Islam and Trapnell datasets contain ∼65*%* and ∼48*%* zeros, respectively. 10x Genomics datasets are known to contain even more zeros. The evaluated subset of the PBMC dataset contains ∼87*%* zeros. The simulated datasets successfully mimic the characteristics of the original datasets, as evaluated with the R package COUNTSIMQC [37] (Additional files 2, 3, and 4). This diverse range of datasets is, therefore, representative of scRNA-seq datasets that occur in practice and it is a suitable basis for method evaluation and comparison.

**Fig. 3:**
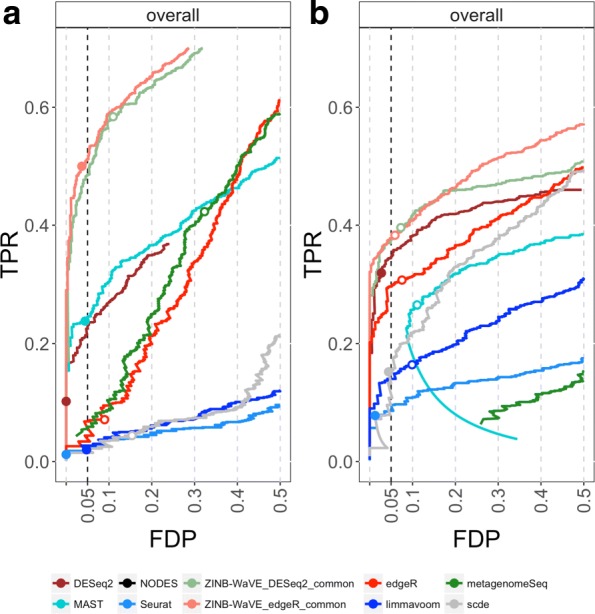
Comparison of differential expression methods on simulated scRNA-seq data. **a** scRNA-seq data simulated from the Islam et al. [16] dataset (*n*=90). **b** scRNA-seq data simulated from the Trapnell et al. [36] dataset (*n*=150). Differential expression methods are compared based on scatterplots of the true positive rate (TPR) vs. the false discovery proportion (FDP). Zoomed versions of the FDP-TPR curves are shown here and the full curves are in Additional file 1: Figure S7. Circles represent working points on a nominal 5% FDR level and are filled if the empirical FDR (i.e., FDP) is below the nominal FDR. Methods based on ZINB-WaVE weights clearly outperform other methods for both simulated datasets. Note that the methods differ in performance between datasets, possibly because of a higher degree of zero inflation in the Islam dataset. The SCDE and METAGENOMESEQ methods, which were specifically developed to deal with excess zeros, are outperformed in both simulations by ZINB-WaVE-based methods and by DESEQ2. The DESEQ2 curve in panel (**a**) is cut off due to not available NA (not available) adjusted *p*-values resulting from independent filtering. The behavior in the lower half of the curve for MAST in (**b**) is due to a smooth increase in true positives with an identical number of false positives over a range of low FDR cut-offs. The curve for NODES is not visible on this figure. It is shown only in the full FDP-TPR curves. FDP false discovery proportion, FDR false discovery rate, TPR true positive rate

We evaluate the performance of the method in terms of sensitivity and false positive control using false discovery proportion vs. true positive rate (FDP-TPR) curves. Figure 3 (Additional file 1: Figure S7) illustrates that many methods break down on the simulated Islam dataset due to a high degree of zero inflation. Surprisingly, even methods specifically developed to deal with excess zeros, like SCDE and METAGENOMESEQ, suffer from poor performance, with MAST being a notable exception. The DESEQ2 methods, however, are able to cope with the high degree of zero inflation. Note that, in general, it is a good strategy to disable the outlier imputation step in DESEQ2, since it deteriorates performance on scRNA-seq data (Additional file 1: Figure S8).

SEURAT, limma-voom, and SCDE have very low sensitivity. The methods based on ZINB-WaVE weights dominate all competitors in terms of sensitivity and specificity, providing high power, good false discovery rate (FDR) control, and sensible *p*-value distributions (Additional file 1: Figure S9). Note that the remaining methods also suffer from poor FDR control.

Since zero inflation is fairly modest for the Trapnell dataset, most methods perform better than for the Islam simulation (Fig. 3). The ZINB-WaVE-based methods and DESEQ2 outperform the remaining methods in terms of sensitivity and provide good FDR control. EDGER is their closest competitor. The remaining methods provide much lower sensitivity and/or very liberal FDR control. Note how bespoke scRNA-seq methods seem to break down on datasets with a lower degree of zero inflation, often providing too liberal or too conservative *p*-value distributions, while ZINB-WaVE-based methods, in general, show a reasonable *p*-value distribution, with an enrichment of low *p*-values and approximately uniformly distributed larger *p*-values (Additional file 1: Figure S10).

**Fig. 4:**
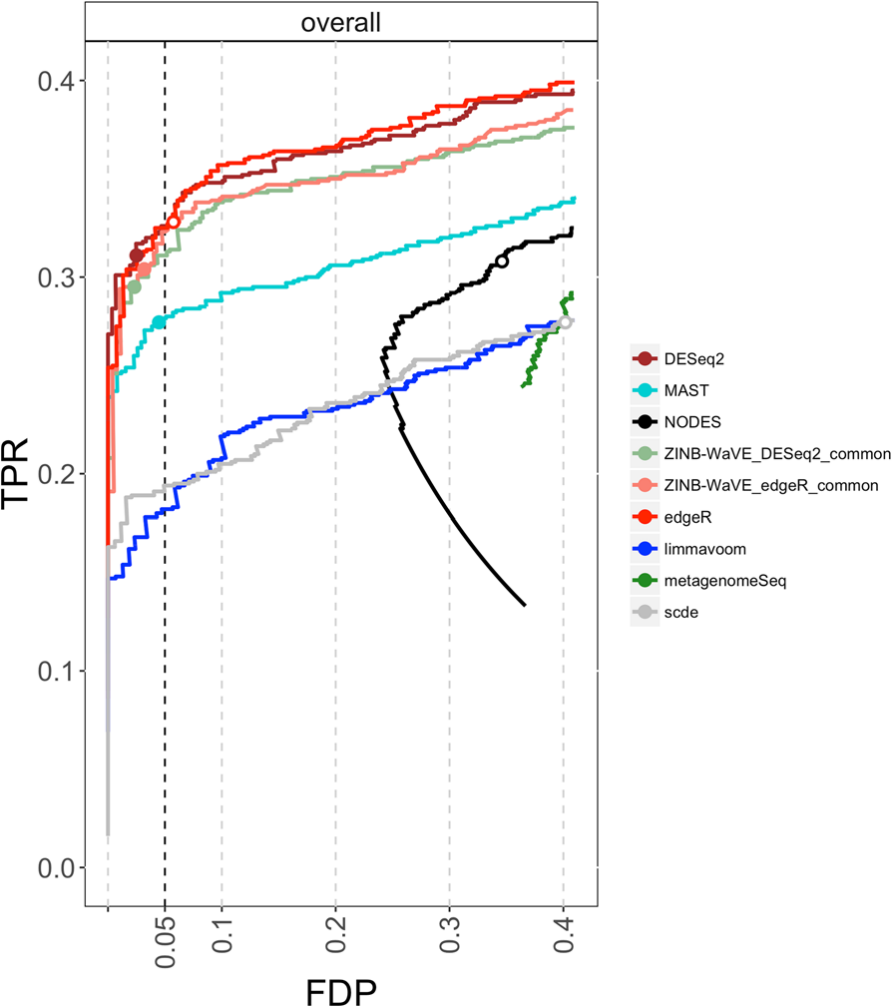
Comparison of differential expression methods on simulated scRNA-seq datasets. Differential expression methods are compared based on FDP-TPR curves for data simulated from a 10x Genomics PBMC single-cell RNA-seq dataset (*n*=1200). Zoomed versions of the FDP-TPR curves are shown here and full curves are in Additional file 1: Figure S12. Circles represent working points on a nominal 5% FDR level and are filled if the empirical FDR (i.e., FDP) is below the nominal FDR. 10x Genomics sequencing typically involves high-throughput and massive multiplexing, resulting in very shallow sequencing depths and thus, low counts, making it extremely difficult to identify excess zeros. Unweighted and ZINB-WaVE-weighted EDGER are tied for best performance, followed by ZINB-WaVE-weighted DESEQ2. In general, bulk RNA-seq methods perform well in this simulation, probably because the extremely high zero abundance in combination with low counts can be reasonably accommodated by the negative binomial distribution. The behavior in the lower half of the curve for NODES is due to a smooth increase in true positives with an identical number of false positives over a range of low FDR cut-offs. FDP false discovery proportion, FDR false discovery rate, PBMC peripheral blood mononuclear cell, TPR true positive rate

Typical 10x Genomics datasets contain a high number of cells with shallow sequencing depth, due to the extreme multiplexing of libraries. As a result, counts and hence, estimated NB means are lower, making zeros more plausible according to the NB distribution and excess zeros, thus, harder to identify. This is picked up by the simulation framework, where only ∼8*%* of the genes were simulated to have at least one excess zero in *n*=1200 samples. Bulk RNA-seq methods can, hence, be expected to be among the top performers. Figure 4 shows FDP-TPR curves for the 10x Genomics simulation study, demonstrating the good performance of bulk RNA-seq methods EDGER and DESEQ2. ZINB-WaVE EDGER and ZINB-WaVE DESEQ2 are among the top performers, having comparable or slightly lower performance compared to their unweighted counterparts. MAST is their closest competitor, providing good sensitivity and FDR control. SCDE, NODES, METAGENOMESEQ, and limma-voom have lower sensitivity and/or very liberal FDR control compared to the dominant methods. These results suggest that, in a scenario of low counts or low degree of zero inflation, ZINB-WaVE-weighted EDGER/DESEQ2 reduce to standard unweighted EDGER/DESEQ2, while other bespoke scRNA-seq tools may deteriorate in performance. This is further supported by results on simulated bulk RNA-seq data, where ZINB-WaVE-weighted EDGER/DESEQ2 have similar performance as standard unweighted EDGER/DESEQ2 in the absence of zero inflation (Additional file 1: Figure S11). Hence, adopting ZINB-WaVE-based DE methods provides a performance boost in zero-inflated applications, while performance is similar in the absence of zero inflation.

All analyses performed in this work are based on estimating one common dispersion parameter across all genes for the ZINB-WaVE model. ZINB-WaVE allows the estimation of genewise dispersion parameters; however, this approach is much more computationally intensive and can be an order of magnitude slower. Additional file 1: Figures S7 and S12 show that estimating genewise dispersion parameters does not seem to be required for calculating the ZINB-WaVE weights, since no gain in performance is achieved when doing so. Note that genewise dispersions are still estimated by EDGER and DESEQ2 in the final DE inference procedure.

### False positive rate control

We compared our ZINB-WaVE-weight-based method to commonly used DE methods for mock comparisons based on two publicly available real scRNA-seq datasets. We assessed performance based on the per-comparison error rate (PCER), defined as the proportion of false positives (i.e., type I errors) among all genes being considered for DE, where a gene is declared DE if its nominal unadjusted *p*-value is less than or equal to 0.05.

**Fig. 5:**
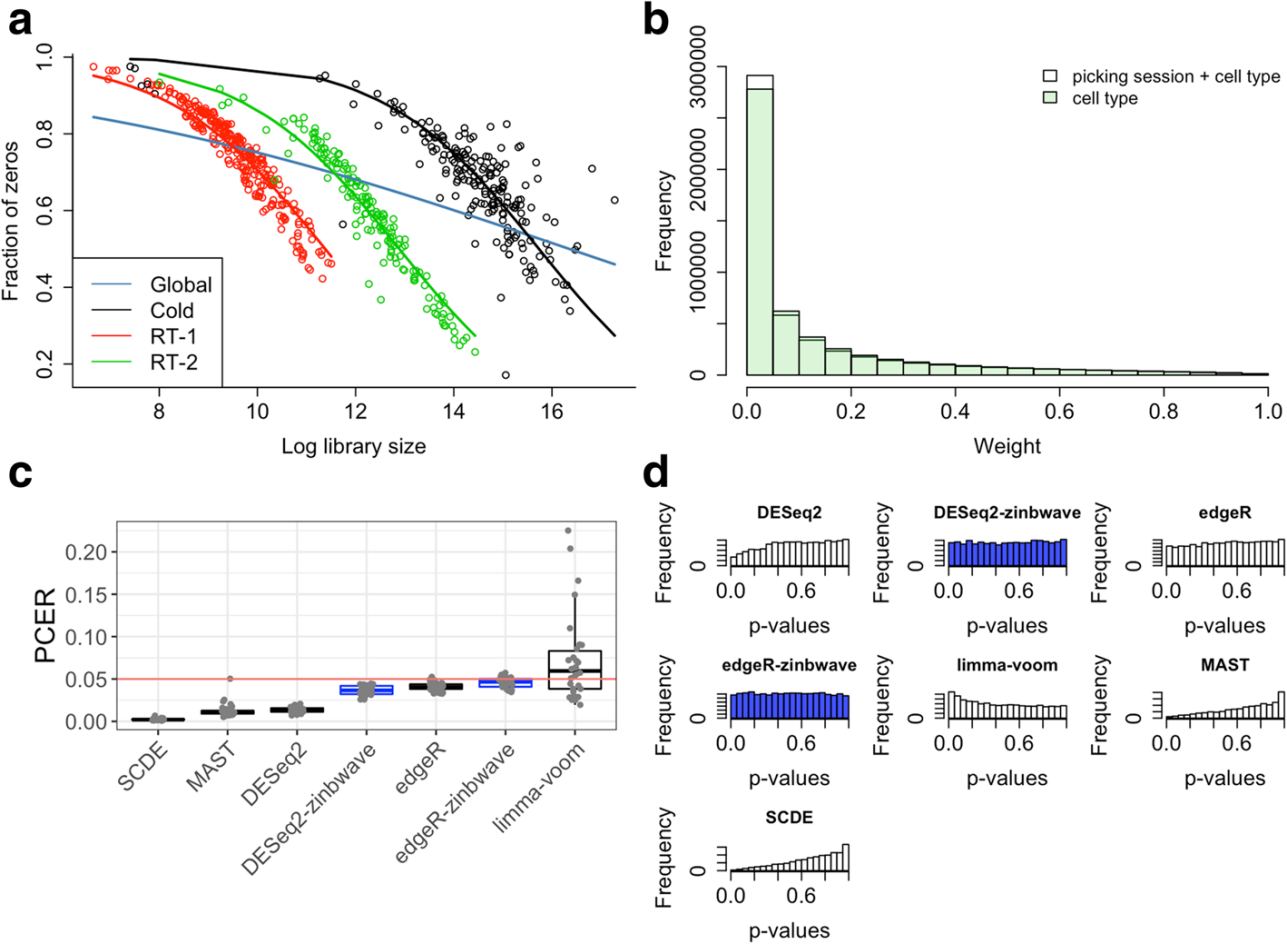
False positive control on mock null Usoskin datasets (*n*=622 cells). **a** The scatterplot and GLM fits (R glm function with family=binomial), color-coded by batch (i.e., picking sessions Cold, RT-1, and RT-2), illustrate the association of zero abundance with sequencing depth. The three batches differ in their sequencing depths, causing an attenuated global relationship when pooling cells across batches (blue curve). Adjusting for the batch effect in the ZINB-WaVE model allows us to account for the relationship between sequencing depth and zero abundance properly. **b** Histogram of ZINB-WaVE weights for zero counts for original Usoskin dataset, with (white) and without (green) including batch as a covariate in the ZINB-WaVE model. The higher mode near zero for batch adjustment indicates that more counts are classified as dropouts, suggesting a more informative discrimination between excess and negative binomial zeros. **c** Box plot of per-comparison error rate (PCER) for 30 mock null datasets for each of seven differential expression methods. ZINB-WaVE-weighted methods are highlighted in blue. **d** Histogram of unadjusted *p*-values for one of the datasets in (**c**). ZINB-WaVE was fitted with the intercept, cell-type covariate (actual or mock), and batch covariate (unless specified otherwise) in *X*, *V*=**1**_*J*_, *K*=0 for *W*, common dispersion, and *ε*=10^12^. GLM generalized linear model, PCER per-comparison error rate

The first dataset, referred to as the Usoskin [11] dataset, is for 622 mouse neuronal cells from the dorsal root ganglion, classified into 11 categories. The authors acknowledge the existence of a batch effect related to the picking session for the cells. We find that the batch effect is not only associated with expression measures, but also influences the relationship between sequencing depth and zero abundance (Fig. 5a) [38]. The large differences in sequencing depths between batches attenuate the overall association with zero abundance when cells are pooled across batches (Fig. 5a). We, therefore, added a covariate to account for the batch effect in both the NB mean (*μ*) and the zero-inflation probability (*π*) of the ZINB-WaVE model used to produce the weights for DE analysis. Adjusting for the batch yields weights with a slightly higher mode near zero, suggesting a more informative discrimination between excess and NB zeros (Fig. 5b). Although the batch effect is small in terms of the weights, this illustrates the generality and flexibility of our ZINB-WaVE weighting approach. With a suitable parameterization of both the NB mean and zero-inflation probability, one can adjust for effects that can bias the weights and hence the DE results.

For the Usoskin dataset, we assessed false positive control by comparing the *actual* vs. the *nominal* PCER for mock null datasets where none of the genes are expected to be DE. Specifically, we generated 30 mock datasets where, for each dataset, two groups of 45 cells each were created by sampling 15 cells at random without replacement from each of the three picking sessions. Sampling cells within batch allows us to control for potential confounding by the batch variable. For each of the 30 mock datasets, we considered seven methods to identify genes that are DE between the two groups and declared a gene DE if its nominal unadjusted *p*-value was less than or equal to 0.05. For these mock datasets, any gene declared DE between the two groups is a false positive. Thus, for each method, the nominal PCER of 0.05 is compared to the actual PCER, which is simply the proportion of genes declared DE (Fig. 5c,d).

The seven methods considered are: unweighted and ZINB-WaVE-weighted EDGER, unweighted and ZINB-WaVE-weighted DESEQ2, unweighted limma-voom (ZINB-WaVE-weighted limma-voom was found to perform poorly in the simulation study and hence, is not considered here), MAST, and SCDE (see “Methods” for details). EDGER and DESEQ2 with ZINB-WaVE weights and unweighted EDGER controlled the PCER close to its nominal level (Fig. 5c). The unweighted versions of DESEQ2, MAST, and SCDE tended to be conservative, whereas limma-voom tended to be anti-conservative. In addition, the weighted versions of EDGER and DESEQ2 and unweighted EDGER yielded near uniform *p*-value distributions (as expected under this complete null scenario), while unweighted DESEQ2, MAST, and SCDE tended to yield conservative *p*-values (mode near 1) and limma-voom yielded anti-conservative *p*-values (mode near 0) (Fig. 5d).

We also replicated the original analysis of Usoskin et al. [11], by performing one-against-others tests of DE for each cell type (Additional file 1: Figure S13). limma-voom found a high number of DE genes, confirming our results from the mock evaluations where it was too liberal. The ZINB-WaVE methods tended to find a high number of DE genes, which is promising combined with the good PCER control seen in the mock comparisons. While introducing ZINB-WaVE weights in DESEQ2 leads to a higher number of significant genes on average, the effect is less clear with EDGER and seems to depend on the contrast.

Similar results were observed for a 10x Genomics PBMC dataset comprising 2700 single cells sequenced on an Illumina NextSeq 500 (Additional file 1: Figure S14), with the distinction that we found a conservative *p*-value distribution for ZINB-WaVE-weighted DESEQ2. Since no information was provided about potential batch effects, we did not consider batch covariates for this dataset.

Additionally, we examined the PCER and *p*-value distributions on mock comparisons while varying the regularization parameter (*ε*) for the ZINB-WaVE estimation procedure. Not surprisingly, we observed that the PCER decreases with increasing *ε*, i.e., as the parameters of the ZINB-WaVE model are subjected to more “shrinking” (Additional file 1: Figures S15 and S16 for the Usoskin and 10x Genomics PBMC datasets, respectively).

### Biologically meaningful clustering and DE results

**Fig. 6:**
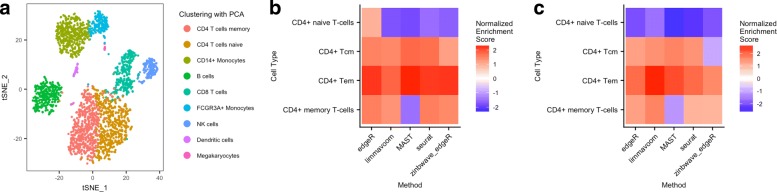
Biologically meaningful DE results for the 10x Genomics PBMC dataset. **a** Scatterplot of the first two t-SNE dimensions obtained from the first ten principal components. Cells are color-coded by clusters found using the SEURAT graph-based clustering method on the first ten principal components. Pseudo-color images on the right display normalized enrichment scores after gene set enrichment analysis for cell types related to CD4+ T cells (see “Methods”), for clustering based on **b** the first ten principal components and **c***W* from ZINB-WaVE with *K*=20. For dimensionality reduction, ZINB-WaVE was fitted with *X*=**1**_*n*_, *V*=**1**_*J*_, *K*=20 for *W* (based on the Akaike information criterion), common dispersion, and *ε*=10^12^. To compute the weights for differential expression analysis, ZINB-WaVE was fitted with intercept and cell-type covariate in *X*, *V*=**1**_*J*_, *K*=0 for *W*, common dispersion, and *ε*=10^12^. Normalized enrichment scores for more cell types are shown in Additional file 1: Figure S17. PCA principal component analysis

To analyze the 2700 cells from the 10x Genomics PBMC dataset (see “Methods”), we followed the tutorial available at http://satijalab.org/seurat/pbmc3k_tutorial.html and used the R package SEURAT [39]. The major steps of the pipeline were quality control, data filtering, identification of high-variance genes, dimensionality reduction using the first ten components from principal component analysis (PCA), and graph-based clustering. The final step of the pipeline was to identify genes that are DE between clusters, to derive cell-type signatures. Two different parameterizations were used for the SEURAT clustering. With one parameterization, a single cluster was identified for CD4+ T cells, while with another, two CD4+ T-cell subclusters were identified, corresponding to CD4+ naive T cells and CD4+ memory T cells (gold and red clusters in Fig. 6a, respectively). At the end of the tutorial, the authors concluded that the memory/naive split was weak and more cells would be needed to give a better separation between the two CD4+ T-cell subclusters.

To find DE genes between the two CD4+ T-cell subclusters, we used SEURAT, unweighted EDGER, ZINB-WaVE-weighted EDGER, MAST, and limma-voom. We then sought to identify cell types using gene set enrichment analysis (GSEA), with the function fgsea from the Bioconductor R package FGSEA [40] and gene sets for 64 immune and stroma cell types from the R package XCELL [41]. While unweighted EDGER found that one cluster was enriched in both CD4+ memory and naive T cells compared to the other cluster, our weighted EDGER method as well as SEURAT and limma-voom found that the cluster was enriched in CD4+ T-effector memory, CD4+ T-central memory, and CD4+ memory T cells, and depleted in CD4+ naive T cells. MAST found that the cluster was depleted in CD4+ memory T cells and CD4+ naive T cells, but enriched in CD4+ T-effector memory and CD4+ T-central memory T cells (see Fig. 6b and Additional file 1: Figure S17). This suggests that our ZINB-WaVE weights can successfully unlock EDGER for zero-inflated data, leading to biologically meaningful DE genes.

While ZINB-WaVE can be used to compute weights in a supervised setting with a priori known cell types, it can also be used to perform dimensionality reduction in an unsupervised setting. To demonstrate the ability of our method to find biologically relevant clusters and DE genes, we performed dimensionality reduction using ZINB-WaVE with *K*=20 unknown covariates (matrix *W*, see “Methods”), where *K*=20 was chosen using the Akaike information criterion (AIC) (Additional file 1: Figure S18). We then used *W*, instead of the first ten components of PCA as in the SEURAT tutorial, to cluster the cells using SEURAT graph-based clustering. We found similar clusters as the SEURAT clusters, except for the NK-cell and B-cell clusters, which were partitioned differently and the cluster with CD4+ T cells (Additional file 1: Figure S19). Using this new clustering, GSEA showed a better separation between CD4+ naive T cells and CD4+ memory T cells for all the methods, suggesting a biologically meaningful clustering using ZINB-WaVE dimensionality reduction instead of PCA. The CD4+ T-effector memory, CD4+ T-central memory, and CD4+ memory cell types were enriched using limma-voom, unweighted EDGER, MAST, and SEURAT, but only the CD4+ T-central memory cell type was depleted using our weighted EDGER method (Fig. 6c and Additional file 1: Figure S17). As we do not have prior knowledge about the cells in the different clusters, we are unable to say whether the cluster is more representative of the CD4+ T-effector memory cell type or if our method missed the enrichment in the CD4+ T-central memory cell type. However, it is interesting that using ZINB-WaVE to account for zero inflation in the clustering allowed EDGER to find results that seem more biologically meaningful than without accounting for zero inflation.

Finally, using a Benjamini and Hochberg [42] adjusted *p*-value cut-off of 0.05, limma-voom declared 433 and 194 DE genes and weighted EDGER 371 and 151, for clustering based on, respectively, the first ten PCs and *W* from ZINB-WaVE. We additionally showed on mock comparisons for the same 10x Genomics PBMC dataset that limma-voom had a greater actual PCER than weighted EDGER (Additional file 1: Figure S14), suggesting that some of the DE genes found by limma-voom are likely to be false positives. This belief is reinforced by the skewed distribution of limma-voom *p*-values (Additional file 1: Figure S20).

### Alternative approaches to weight estimation

ZINB-WaVE is one particular approach for fitting a ZINB model to scRNA-seq data. However, our proposed data analysis strategy for unlocking conventional RNA-seq tools with ZINB observation-level weights is not restricted to ZINB-WaVE-based workflows. In particular, we illustrate the use of weights estimated by the ZINGER method, an expectation-maximization algorithm, which we developed earlier and which builds upon EDGER for estimating the NB parameters of the ZINB model [43]. The ZINB-WaVE and ZINGER approaches differ in the following respects. The ZINGER weights are based on a constant cell-specific excess zero probability *π*_*i*_ for each cell *i*, while the ZINB-WaVE excess zero probability *π*_*ij*_ is both cell- and gene-specific, a strategy that has also been advocated in recent methods [19, 22]. Secondly, the ZINB-WaVE NB mean *μ* and zero-inflation probability *π* are modeled in terms of both wanted and unwanted cell- and gene-level covariates, allowing normalization for a variety of nuisance technical effects. Thirdly, different parameter estimation strategies are adopted. Parameters from the ZINGER model are estimated with an expectation-maximization algorithm, whereas those from the ZINB-WaVE model are estimated using a penalized maximum likelihood approach. Finally, methods based on ZINGER weights have the property of converging to their unweighted counterparts in the absence of zero inflation.

In terms of performance, based on the simulation study on full-length protocols, ZINGER workflows dominate both bulk RNA-seq and dedicated scRNA-seq methods, but were found to be inferior in terms of sensitivity to ZINB-WaVE workflows (Additional file 1: Figure S21). However, for the Usoskin dataset, ZINGER seems to find a higher number of DE genes than ZINB-WaVE and its bulk RNA-seq counterparts (Additional file 1: Figure S22), while also controlling the PCER in mock evaluations (Additional file 1: Figure S23). Due to the computational burden of the ZINGER method, we were unable to apply it to large-scale datasets, such as those from the 10x Genomics platform, thus limiting our comparison.

### Computational time

The better performance of our ZINB-WaVE-weighted DE method comes at a computational cost, since we first fit ZINB-WaVE to the entire cells-by-genes matrix of read counts to compute the weights and then use a weighted version of DESEQ2 or EDGER for inferring DE. To give the reader an idea of how different methods scale in terms of computation time, we benchmarked three different datasets: the Islam dataset (92 cells), one of the mock null Usoskin datasets used in Fig. 5 (90 cells), and the CD4+ T-cell cluster of the 10x Genomics PBMC dataset (1151 cells). For each dataset, 10,000 genes were sampled at random and the two cell types were used as covariates. For the Usoskin dataset, batch was added as a covariate for all methods. For all datasets, the fastest method was limma-voom followed by EDGER (Additional file 1: Figure S24). As DESEQ2 was slower than EDGER, not surprisingly weighted DESEQ2 was also slower than weighted EDGER, especially for the 10x Genomics PBMC dataset.

## Discussion

This manuscript focused on adapting standard bulk RNA-seq DE tools to handle the severe zero inflation present in scRNA-seq data. We proposed a simple and general approach that integrates seamlessly with a range of popular DE software packages, such as EDGER and DESEQ2. The main idea is to use weights for zero inflation in the NB model underlying bulk RNA-seq methods. In particular, the weights are based on the ZINB-WaVE method of Risso et al. [23]. The general and flexible ZINB-WaVE framework allows us to extract a low-dimensional signal from scRNA-seq read counts, accounting for zero inflation (e.g., dropouts), over-dispersion, and the discrete nature of the data. In particular, the ZINB-WaVE model allows for read count normalization through an appropriate parameterization of the NB means and zero-inflation probabilities in terms of both gene- and cell-level covariates.

Our results complement the findings of Jaakkola et al. [29] and Soneson and Robinson [30] that bespoke scRNA-seq tools do not systematically improve upon bulk RNA-seq tools. Although MAST, METAGENOMESEQ, and SCDE were explicitly developed to handle excess zeros, they suffer from poor performance in a high zero-inflation setting, as demonstrated in the simulation study.

The value of our method was demonstrated for scRNA-seq protocols relying on both standard (Islam, Usoskin, and Trapnell datasets) and unique molecular identifier (UMI) (10x Genomics PBMC dataset) read counting. UMIs were recently proposed to reduce measurement variability across samples [15]. In UMI-based protocols, transcripts are labeled with a small random UMI barcode prior to amplification. After amplification and sequencing, one enumerates the unique UMIs found for every transcript, which correspond to individual sequenced UMI-labeled transcripts. There is some evidence in the literature that zero inflation is less of a problem for UMI-based than for full-length protocols and that UMI read counts could follow a NB distribution [44, 45]. Hence, our method also provides good results for UMI-based data with limited zero inflation, demonstrating its broad applicability.

In the simulation study, power to detect DE was generally lower for 10x Genomics UMI datasets (Fig. 4) than for full-length protocol datasets (Fig. 3). While the 10x Genomics platform has the advantage of an extremely high throughput, allowing many cells to be characterized, the resulting datasets often have the disadvantage of low library sizes, a logical consequence of UMI counting and of the trade-off between sequencing depth and number of cells to be sequenced in one sequencing run. As a result, the sequencing depth of these datasets is much lower than that of bulk RNA-seq datasets, making it harder to identify excess zeros and assess DE, even in large sample size settings. Although the 10x Genomics platform may be well suited for hypothesis generation, e.g., through cell-type discovery or lineage trajectory studies, full-length protocols may be more appropriate for discovering marker genes between inferred cell types or trajectories, an approach that has also been adopted in previous studies [46].

We have used ZINB-WaVE in conjunction with either EDGER or DESEQ2. However, the ZINB-WaVE posterior probabilities could be used as weights to unlock other standard RNA-seq workflows in zero-inflation situations. Additional file 1: Figure S7 shows that ZINB-WaVE weights combined with heteroscedastic weights in limma-voom also increase power in a scRNA-seq context, although this may be at the expense of type I error control.

The ZINB-WaVE method penalizes the L2 norm of the parameter estimates for regularization purposes. It requires a penalty parameter *ε* that is rescaled differently for gene-specific parameters, cell-specific parameters, and dispersion parameters [23]. All analyses in this manuscript were performed with *ε*=10^12^, to provide consistently comparable results. However, the optimal value of *ε* is dataset-specific and further research is needed to provide a data-driven approach for selecting an optimal *ε*. Indeed, based on our simulations, the value of the penalty parameter can have a profound influence on the results (Additional file 1: Figure S25), but we found *ε*=10^12^ to have generally good performance.

ZINB-WaVE has an option to infer latent variables *W*, which may correspond to either unmeasured confounding covariates or unmeasured covariates of interest. The observational weights were computed with the number of unknown covariates *K*=0, i.e., no latent variables were inferred. To cluster the real datasets, we inferred an optimal choice of *K* using the AIC (Additional file 1: Figure S18). However, further investigation is needed to confirm that the AIC is appropriate for selecting *K*.

In principle, our proposed ZINB-WaVE model could also be used to identify DE genes both in terms of the NB mean and the zero-inflation probability, reflecting, respectively, a continuum in DE and a more binary (i.e., presence or absence) DE pattern. In this context, the parameters of interest are regression coefficients *β* corresponding to known sample-level covariates in the matrix *X* used in either *μ* or *π* (Eq. 4). DE genes may be identified via likelihood ratio tests or Wald tests, with the standard errors of estimators of *β* obtained from the inverse of the Hessian matrix of the likelihood function. However, both types of tests would be computationally costly, as likelihood ratio tests would require refitting the entire model for each gene and Wald tests would require the Hessian matrix to be computed and inverted.

In this contribution, we have proposed estimating the weights using ZINB-WaVE, but other approaches are possible. It is important to note that while methods such as ZINB-WaVE and ZINGER can successfully identify excess zeros, they cannot, however, readily discriminate between their underlying causes, i.e., between technical (e.g., dropout) and biological (e.g., bursting) zeros. Although we cannot make this distinction with the weights, an increase in bursting rates between cell types, characterized by higher counts and more zeros [47], can, however, be picked up by the count component of the ZINB model.

## Conclusion

In summary, we provide a realistic simulation framework for scRNA-seq data and use the well-tested ZINB-WaVE method to identify excess zeros successfully and yield gene- and cell-specific weights for DE analysis in scRNA-seq experiments. The tools we have developed allow an integrated workflow for normalization, dimensionality reduction, cell-type discovery, and the identification of cell-type marker genes. We confirmed that state-of-the-art scRNA-seq tools do not improve upon common bulk RNA-seq tools for DE analysis based on scRNA-seq data. Our workflow, however, outperforms current methods and has the merit that its performance does not deteriorate in the absence of zero inflation. The inference of DE is focused on the count component of the ZINB model and our method produces posterior probabilities that can be used as observation-level weights by bulk RNA-seq tools. Hence, our approach unlocks widely used bulk RNA-seq DE workflows for zero-inflated data and will assist researchers, data analysts, and developers in improving power to detect DE in the presence of excess zeros. The framework is general and applicable beyond scRNA-seq, to zero-inflated count data structures arising in applications such as metagenomics [48, 49].

## Methods

### ZINB-WaVE: Zero-inflated negative binomial-based wanted variation extraction

#### Zero-inflated distributions

A major difference between single-cell and bulk RNA-seq data is arguably the high abundance of zero counts in the former. Traditionally, excess zeros are dealt with using hurdle or zero-inflated models, as recently proposed by Finak et al. [19], Kharchenko et al. [28], and Paulson et al. [48]. A zero-inflated count distribution is a two-component mixture distribution between a point mass at zero and a count distribution, in our case, the NB distribution, which has been used successfully for bulk RNA-seq [1–3, 50].

The probability mass function *f*_ZINB_ for the ZINB distribution is given by

3 $${} {{\begin{aligned} f_{\text{ZINB}}\left(y; \mu, \theta, \pi\right) = \pi \delta_{0}(y) + \left(1-\pi\right) f_{\text{NB}}\left(y; \mu, \theta\right), \quad \forall y\in\mathbb{N}, \end{aligned}}}  $$

where *π*∈[0,1] denotes the mixture probability for zero inflation, *f*_NB_(·;*μ*,*θ*) the NB probability mass function with mean *μ* and dispersion *θ*=1/*ϕ*, and *δ*_0_(·) the Dirac function [ *δ*_0_(*y*)=+*∞* when *y*=0 and 0 otherwise and *δ*_0_ integrates to one over $\mathbb {R}$, i.e., has cumulative distribution function equal to *I*(*y*≥0)]. Here, *π* can be interpreted as the probability of an excess zero, i.e., inflated zero count, with respect to the NB distribution.

Under a ZINB model, the posterior probability that a given count *y* arises from the NB count component is given by Bayes’ rule:

$$w = \frac{\left(1 - \pi\right) f_{\text{NB}}\left(y; \mu, \theta \right) }{f_{\text{ZINB}}\left(y;\mu, \theta, \pi\right)}. $$

As described below, such posterior probabilities can be used as weights in standard bulk RNA-seq workflows, for a suitable parameterization of the zero-inflation probability and NB mean.

#### ZINB-WaVE model

Given *n* observations (typically, *n* single cells) and *J* features (typically, *J* genes) that can be counted for each observation, let *Y*_*ij*_ denote the count of feature *j* (*j*=1,…,*J*) for observation *i* (*i*=1,…,*n*). To account for various technical and biological effects frequent in single-cell sequencing technologies, we model *Y*_*ij*_ as a random variable following a ZINB distribution with parameters *μ*_*ij*_,*θ*_*ij*_, and *π*_*ij*_, and consider the following regression models for these parameters:

4 $$\begin{array}{*{20}l} \ln\left(\mu_{ij}\right) &= \left(X\beta_{\mu} + \left(V\gamma_{\mu}\right)^{\top} + W\alpha_{\mu} + O_{\mu}\right)_{ij},\\ \text{logit}\left(\pi_{ij}\right) &= \left(X\beta_{\pi} + \left(V\gamma_{\pi}\right)^{\top} + W\alpha_{\pi} + O_{\pi}\right)_{ij}, \\ \ln(\theta_{ij}) &= \zeta_{j}.  \end{array} $$

Both the NB mean expression level *μ* and the zero-inflation probability *π* are modeled in terms of *observed sample-level and gene-level covariates* (*X* and *V*, respectively), as well as *unobserved sample-level covariates* (*W*) that need to be inferred from the data. *O*_*μ*_ and *O*_*π*_ are known matrices of offsets. The matrix *X* can include covariates that induce a variation of interest, such as cell types, or covariates that induce unwanted variation, such as batch or quality control measures. It can also include a constant column of ones for an intercept that accounts for gene-specific global differences in mean expression level or dropout rate. The matrix *V* can include gene-level covariates, such as length or GC content. It can also accommodate an intercept to account for cell-specific global effects, such as size factors representing differences in library sizes (i.e., total number of reads per sample). The unobserved matrix *W* contains unknown sample-level covariates, which could correspond to unwanted variation as in RUV [33, 34] (e.g., a priori unknown batch effects) or could be of interest as in cluster analysis (e.g., a priori unknown cell types). The model extends the RUV framework to the ZINB distribution (thus far, RUV had only been implemented for linear [33] and log-linear regression [34]). It differs, however, in interpretation from RUV in the *Wα* term, which is not necessarily considered unwanted and generally refers to unknown low-dimensional variation. It is important to note that although *W* is the same, the matrices *X* and *V* could differ in the modeling of *μ* and *π*, if we assume that some known factors do not affect both.

As detailed in Risso et al. [23], the model is fitted using a penalized maximum likelihood estimation procedure.

### Using ZINB-WaVE weights in DE inference methods

We consider only statistical inference on the count component of the mixture distribution, that is, we are concerned with identifying genes whose expression levels are associated with covariates of interest as parameterized in the mean *μ* of the NB component. Most popular bulk RNA-seq methods are based on the methodology of GLMs, which readily accommodates inference based on observation-level weights (R function glm), e.g., the NB model in Bioconductor R packages EDGER and DESEQ2. Note that although the ZINB-WaVE weights are gene- and cell-specific, the GLMs are fitted gene by gene. Hence, for a given gene, the cell-specific weights are used as observation-specific weights in the GLMs.

#### EDGER

We extended the EDGER package [2, 50] by fitting a NB model genewise, with ZINB-WaVE posterior probabilities as observation-level weights in the weights slot of an object of class *DGEList*, and estimating the dispersion parameter by the usual approximate empirical Bayes shrinkage. Downweighting is accounted for by adjusting the degrees of freedom of the null distribution of the test statistics. Specifically, we reintroduced the moderated *F* test from a previous version of EDGER, where the denominator residual degrees of freedom *df*_*j*_ for a particular gene *j* are adjusted by the extent of zero inflation identified for this gene, i.e., $df_{j}=\sum _{i} w_{ij} - p$, where *w*_*ij*_ is the ZINB-WaVE weight for gene *j* in cell *i* and *p* the number of parameters estimated in the NB GLM. This weighted *F* test is implemented in the function glmWeightedF from the Bioconductor R package ZINBWAVE.

#### DESEQ2

We extended the DESEQ2 package [1] to accommodate zero inflation by providing the option to use observation-level weights in the parameter estimation steps. In this case, the ZINB-WaVE weights are supplied in the weight slot of an object of class *DESeqDataSet*.

DESEQ2’s default normalization procedure is based on geometric means of counts, which are zero for genes with at least one zero count. This greatly limits the number of genes that can be used for normalization in scRNA-seq applications [51]. We, therefore, use the normalization method suggested in the PHYLOSEQ package [52], which calculates the geometric mean for a gene using only its positive counts, so that genes with zero counts could still be used for normalization. The PHYLOSEQ normalization procedure can now be applied by setting the argument type equal to poscounts in the DESEQ2 function estimateSizeFactors. For single-cell UMI data, for which the expected counts may be very low, the likelihood ratio test implemented in nbinomLRT should be used. For other protocols (i.e., non-UMI), the Wald test in nbinomWaldTest can be used, with null distribution a *t* distribution with degrees of freedom corrected for downweighting. In both cases, we recommend the minimum expected count to be set to a small value (minmu=1e-6). The Wald test in DESEQ2 allows for testing contrasts of the coefficients.

#### limma-voom

For the limma-voom approach [3], implemented in the voom function from the LIMMA package, heteroscedastic weights are estimated based on the mean–variance relationship of the log-transformed counts. We adapt limma-voom to zero-inflated situations by multiplying the heteroscedastic weights by the ZINB-WaVE weights and using the resulting weights in weighted linear regression. To account for the downweighting of zeros, the residual degrees of freedom of the linear model are adjusted, such as with EDGER, before the empirical Bayes variance shrinkage and are, therefore, also propagated to the moderated statistical tests. Both the standard and ZINB-WaVE-weighted versions of limma-voom were considered in the simulation study. The latter was not considered for the real datasets due to its poor performance in the simulation study.

#### Multiple testing

For the simulation study, to reduce the number of tests performed [53], we apply the independent filtering procedure implemented in the GENEFILTER package and used in DESEQ2 [1]. As in DESEQ2, we exclude from the multiple testing correction any gene whose average expression strength (i.e., average of fitted values) is below a threshold chosen to maximize the number of DE genes. Note that the filtering procedure can affect each method differently, due to differences in fitted values and *p*-value distributions. Unless specified otherwise, the *p*-values for all methods are then adjusted using the Benjamini and Hochberg [42] procedure for controlling the FDR.

#### Performance assessment

We assess performance based on scatterplots of the TPR vs. the FDP, as well as receiver operating characteristic (ROC) curves of the TPR vs. the false positive rate (FPR), according to the following definitions

$$\begin{array}{*{20}l} \text{FDP} &= \frac{\text{FP}}{\max(1,\text{FP} + \text{TP})}\\ \text{FPR} &= \frac{\text{FP}}{\text{FP} + \text{TN}}\\ \text{TPR} &= \frac{\text{TP}}{\text{TP} + \text{FN}}, \end{array} $$

where FN, FP, TN, and TP denote, respectively, the numbers of false negatives, false positives, true negatives, and true positives. FDP-TPR curves and ROC curves are implemented in the Bioconductor R package ICOBRA [54].

#### DE method comparison

We compared our weighted DE approach to state-of-the-art bulk RNA-seq methods implemented in the packages EDGER (v3.20.1) [2, 50], DESEQ2 (v1.19.8) [1], and LIMMA (v3.34.0) [3]. We also considered dedicated scRNA-seq tools from the packages SCDE (v2.6.0) [28], MAST (v1.4.0) [19], and NODES (v0.0.0.9010) [55], as well as METAGENOMESEQ (v1.18.0) [48], which was developed to account for zero inflation in metagenomics applications. A ZINB model is also implemented in SHRINKBAYES [56], but the method does not scale to the typical sample sizes encountered in scRNA-seq and has many tuning parameters, so we did not include it in our comparison. In DESEQ2, we disable the outlier imputation step and allow for shrinkage of fold-changes by default. In addition, for large 3^′^-end sequencing datasets like the Usoskin and 10x Genomics PBMC datasets, we set the minimum expected count estimated by DESEQ2 to 10^−6^, allowing the method to cope with large sample sizes and low counts. We use the recommended gene-filtering procedures for NODES and MAST, except for the computing time benchmark, where no genes are filtered out to allow a fair comparison. For all other methods, arguments were set to their default values.

### scRNA-seq data simulation

We extended the framework of Zhou et al. [57] for scRNA-seq applications. In the GitHub repository linked to this manuscript (https://github.com/statOmics/zinbwaveZinger), we provide user-friendly R code to simulate scRNA-seq read counts. The user can input a real scRNA-seq dataset to infer gene-level parameters for read count distributions. Library sizes for the simulated samples are by default resampled from the real dataset, but can also be user-specified. The simulation paradigm randomly resamples parameters estimated from the original dataset, where all parameters of a given gene are resampled jointly to retain gene-specific characteristics present in the original dataset.

In scRNA-seq, dropouts and bursting lead to bias in parameter estimation if not properly accounted for. Our simulation framework alleviates this problem by using zero-truncated negative binomial (ZTNB) method-of-moments estimators [58, 59] on the positive counts to estimate the expression fraction *λ*_*j*_=*E*[*Y*_*ij*_/*N*_*i*_], with $N_{i} = \sum _{j} Y_{ij}$ the sequencing depth of cell *i*, and the NB dispersion *θ*_*j*_=1/*ϕ*_*j*_. Specifically, initial NB-based estimators are iteratively updated according to the ZTNB-based estimators provided by

5 $${} {{\begin{aligned} \hat\lambda_{j}^{\text{new}} &= \frac{\sum_{i} Y_{ij} \left (1-f_{\text{NB}}\left(0 ; \hat \lambda_{j} N_{i}, \hat \theta_{j}\right) \right)}{\sum_{i} N_{i}},\\ \hat\theta_{j}^{\text{new}} &= \frac{\sum_{i}\left(\hat\lambda_{j} N_{i}\right)^{2}}{\sum_{i} Y_{ij}^{2} \left(1-f_{\text{NB}}\left(0; \hat\lambda_{j} N_{i}, \hat\theta_{j}\right) \right) - \sum_{i} \left(\hat\lambda_{j} N_{i}\right)^{2} - \sum_{i} \left(\hat\lambda_{j} N_{i}\right)}. \end{aligned}}}  $$

Note that, when *Y*_*ij*_ is zero, it does not contribute to the estimators of *λ*_*j*_ and *θ*_*j*_. These estimates are then used to simulate counts according to a NB distribution.

We additionally simulate excess zeros by modeling the empirical zero abundance *p*_*ij*_=*I*(*Y*_*ij*_=0) as a function of an interaction between the gene-specific expression intensity, measured as average log CPM:

$$\hat A_{j} \approx \log_{2} \frac{10^{6}}{n} \sum_{i=1}^{n} \frac{Y_{ij}}{N_{i}} $$

(as calculated using the aveLogCPM function from EDGER), and the cell-specific sequencing depth *N*_*i*_, using a semi-parametric additive logistic regression model:

6 $$\begin{array}{*{20}l}  p_{ij} &\sim B\left(\rho_{ij}\right),\\ \ln \left (\frac{\rho_{ij}}{1-\rho_{ij}} \right) &= s\left(\hat A_{j}\right) + \ln\left(N_{i}\right) + s\left(\hat A_{j}\right) \times \ln\left(N_{i}\right), \end{array} $$

where *B*(*ρ*_*ij*_) denotes the Bernoulli distribution with parameter *ρ*_*ij*_ and *s*(·) is a non-parametric thin-plate spline [60]. We then compare, for every gene, the estimated probability of zero counts based on the model in Eq. 6 to the corresponding NB-based probability $f_{\text {NB}}\left (0;\hat \mu _{ij}, \hat \theta _{j}\right)$ with $\hat \mu _{ij} = \hat \lambda _{j} N_{i}$, and randomly add excess zeros whenever the former probability is higher than the latter. The model in Eq. 6 is motivated by dataset-specific associations observed in real scRNA-seq datasets (Additional file 1: Figures S4, S5).

This framework acknowledges both gene-specific characteristics as well as broad dataset-specific associations across all genes and provides realistic scRNA-seq data for evaluating methods. We assessed the performance of various DE methods using data simulated based on the Islam et al. [16] dataset, a subset of the Trapnell et al. [36] dataset, and a 10x Genomics PBMC dataset. See “Real datasets” for information on these datasets.

### Gene set enrichment analysis

To identify cell types corresponding to the two CD4+ T-cell subclusters of the 10x Genomics PBMC dataset, we used GSEA with the function fgsea from the Bioconductor R package FGSEA (v1.4.0) [40] and gene sets for 64 immune and stroma cell types from the R package XCELL (v1.1.0) [41]. For each DE method, the input to fgsea is a list of genes ranked by a test statistic comparing expression in the two CD4+ T-cell subclusters.

To facilitate comparison between DE methods, the test statistic used here is a transformation of the unadjusted *p*-values (*p*) with the sign of the log-fold-change (lfc): *Φ*^−1^(1−*p*/2) sign(lfc), where *Φ*(·) denotes the standard Gaussian cumulative distribution function. As suggested by FGSEA, all genes were used for the analysis. To assess the enrichment/depletion of one cluster compared to the other cluster, we used the normalized enrichment score. The enrichment score is the same as in the broad GSEA implementation [61] and reflects the degree to which a gene set is overrepresented at the top or bottom of a ranked list of genes. Briefly, the enrichment score is calculated by walking down the ranked list of genes, increasing a running-sum statistic when a gene is in the gene set and decreasing it when it is not. A positive enrichment score indicates enrichment at the top of the ranked list; a negative enrichment score indicates enrichment at the bottom of the ranked list. The enrichment score is then normalized by the mean enrichment of random samples of genes, where genes are permuted from the original ranked list (10,000 permutations were used).

### Real datasets

#### Usoskin dataset

This dataset is for mouse neuronal cells from the dorsal root ganglion, sequenced on either an Illumina Genome Analyzer IIx or HiSeq 2000 [11]. The cells were robotically picked in three separate sessions and the 5^′^ end of the transcripts sequenced. The expression measures were downloaded from supplementary data accompanying the original manuscript (http://linnarssonlab.org/drg/). After quality control and sample filtering (removal of non-single cells and non-neuronal cells), the authors considered 622 cells, which were classified into 11 neuronal cell-type categories. Only genes with more than 20 non-zero counts were retained, for a total of 12,132 genes.

There is a batch effect related to the picking session for the cells. For the DE analysis, the picking session was, therefore, included as a batch covariate in all models.

To mimic a null dataset with no DE, we created two groups of 45 cells each, where, for each group, 15 cells were sampled at random, without replacement (over all cell types) from each picking session. For each of 30 such mock null datasets, we considered seven methods to identify genes that are DE between the two groups and declared a gene DE if its nominal unadjusted *p*-value was less than or equal to 0.05. For these mock datasets, any gene declared DE between the two groups is a false positive. The *nominal* PCER of 0.05 for each method is compared to its *actual* PCER, which is simply the proportion of genes declared DE.

#### 10x Genomics PBMC dataset

We analyzed a dataset of PBMCs that is freely available from 10x Genomics (https://support.10xgenomics.com/single-cell-gene-expression/datasets/1.1.0/pbmc3k) [62]. We downloaded the data from https://s3-us-west-2.amazonaws.com/10x.files/samples/cell/pbmc3k/pbmc3k_filtered_gene_bc_matrices.tar.gz, which correspond to 2700 single cells sequenced on an Illumina NextSeq 500 using UMIs. We clustered cells following the tutorial available at http://satijalab.org/seurat/pbmc3k_tutorial.html and using the R package SEURAT (v2.1.0) [39]. The major steps of the pipeline are quality control, data filtering, identification of high-variance genes, dimensionality reduction using the first ten components from PCA, and graph-based clustering. To identify cluster markers, we used our ZINB-WaVE-weighted DE method instead of the method implemented in SEURAT.

We created 30 mock null datasets and identified DE genes in these as for the Usoskin dataset, i.e., we created two groups of 45 cells each, by sampling at random, without replacement from the 2700 cells of the real dataset (no batch information available).

#### Islam dataset

The count table for the Islam et al. [16] dataset was downloaded from the Gene Expression Omnibus with accession number GSE29087 [35]. The Islam dataset represents 44 embryonic fibroblasts and 48 embryonic stem cells in the mouse, sequenced on an Illumina Genome Analyzer II. Negative control wells were removed and only the 11,796 genes with at least five positive counts were retained for analysis. For the simulation, we generated datasets with two groups of 40 cells each.

#### Trapnell dataset

The dataset from Trapnell et al. [36] was downloaded from the preprocessed single-cell data repository CONQUER (http://imlspenticton.uzh.ch:3838/conquer). Cells were sequenced on either an Illumina HiSeq 2000 or HiSeq 2500. We used only the subset of cells corresponding to the 48 h and 72 h timepoints of differentiating human myoblasts to generate two-group comparisons. Wells that did not contain one cell or that contained debris were removed. We used a more stringent gene-filtering criterion than for the Islam dataset and retained the 24,576 genes with at least ten positive counts. The simulated datasets contain two conditions with 75 cells in each condition, thereby replicating the sample sizes of the Trapnell dataset.

### Software implementation

An R software package for our novel scRNA-seq simulation framework is available from the GitHub repository for this manuscript (https://github.com/statOmics/zinbwaveZinger). Additionally, all analyses and figures reported in the manuscript can be reproduced using code in this GitHub repository. The ZINB-WaVE weight computation is implemented in the computeObservationalWeights function of the Bioconductor R package ZINBWAVE. ZINB-WaVE-weighted EDGER can be implemented using the glmWeightedF function from the ZINBWAVE package, while ZINB-WaVE-weighted DESEQ2 can be implemented using the native nbinomWaldTest function from the DESEQ2 package. More details of the ZINB-WaVE-weighted analysis can be found in the ZINBWAVE vignette (http://bioconductor.org/packages/zinbwave/).

## Additional files

Additional file 1 Supplementary figures. This file contains all supplementary figures to the manuscript. (PDF 8839 kb)

Additional file 2 countsimQC evaluation of simulated Islam dataset. (HTML 11,492 kb)

Additional file 3 countsimQC evaluation of simulated Trapnell dataset. (HTML 11,639 kb)

Additional file 4 countsimQC evaluation of simulated 10x dataset. (HTML 9933 kb)
