# Supplementary material for: Observation weights unlock bulk RNA-seq tools for zero inflation and single-cell applications
Source: Genome Biol. 2018 Feb 26;19:24. doi: 10.1186/s13059-018-1406-4 (PMC6251479; doi:10.1186/s13059-018-1406-4)
Supplement: Supplementary file 1 — Supplementary figures. This file contains all supplementary figures to the manuscript. (PDF 8839 kb) [file 13059_2018_1406_MOESM1_ESM.pdf]

Supplementary Figures to Unlocking bulk RNA-seq tools for zero inflation and single-cell applications using observation weights

Koen Van den Berge[1,2,#], Fanny Perraudeau[3,#], Charlotte Soneson[4,5], Michael I. Love[6], Davide Risso[7], Jean-Philippe Vert[8,9,10,11], Mark D. Robinson[4,5], Sandrine Dudoit[3,12,+,*], Lieven Clement[1,2,+,*]

# Equal contributor, + Equal contributor,
[1] Department of Applied Mathematics, Computer Science and Statistics, Ghent University
[2] Bioinformatics Institute Ghent, Ghent University
[3] Division of Biostatistics, School of Public Health, University of California, Berkeley
[4] Institute of Molecular Life Sciences, University of Zurich
[5] SIB Swiss Institute of Bioinformatics, University of Zurich
[6] Department of Biostatistics and Genetics, The University of North Carolina at Chapel Hill
[7] Division of Biostatistics and Epidemiology, Department of Healthcare Policy and Research, Weill Cornell Medicine
[8] MINES ParisTech, PSL Research University, CBIO-Centre for Computational Biology
[9] Institut Curie, Paris
[10] INSERM U900, Paris
[11] Ecole Normale Supérieure, Department of Mathematics and Applications
[12] Department of Statistics, University of California, Berkeley
∗ Correspondence: sandrine@stat.berkeley.edu, lieven.clement@ugent.be

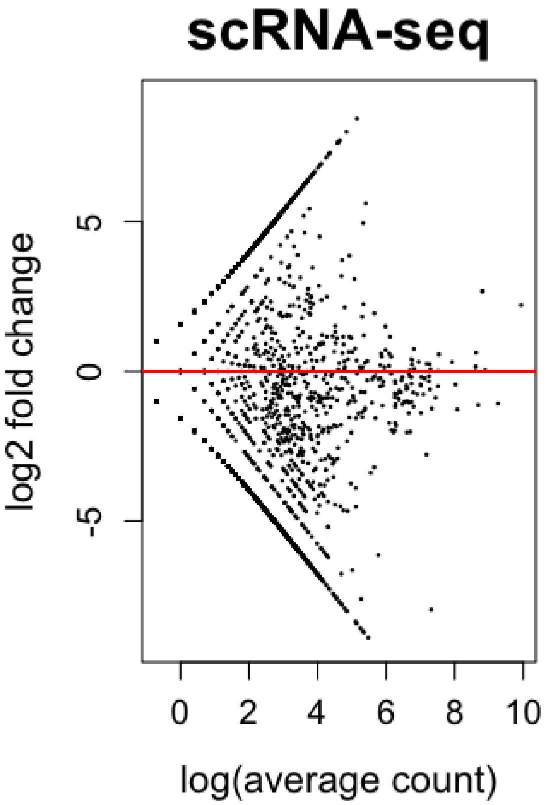

Figure S1: Variability in bulk and single-cell RNA-seq data. Mean-difference plots for two samples from the Islam et al. [2011] scRNA-seq dataset (left panel) and two samples from the Pickrell et al. [2010] bulk RNA-seq dataset (right panel). A higher variability in the scRNA-seq data is observed as compared to the bulk RNA-seq data.

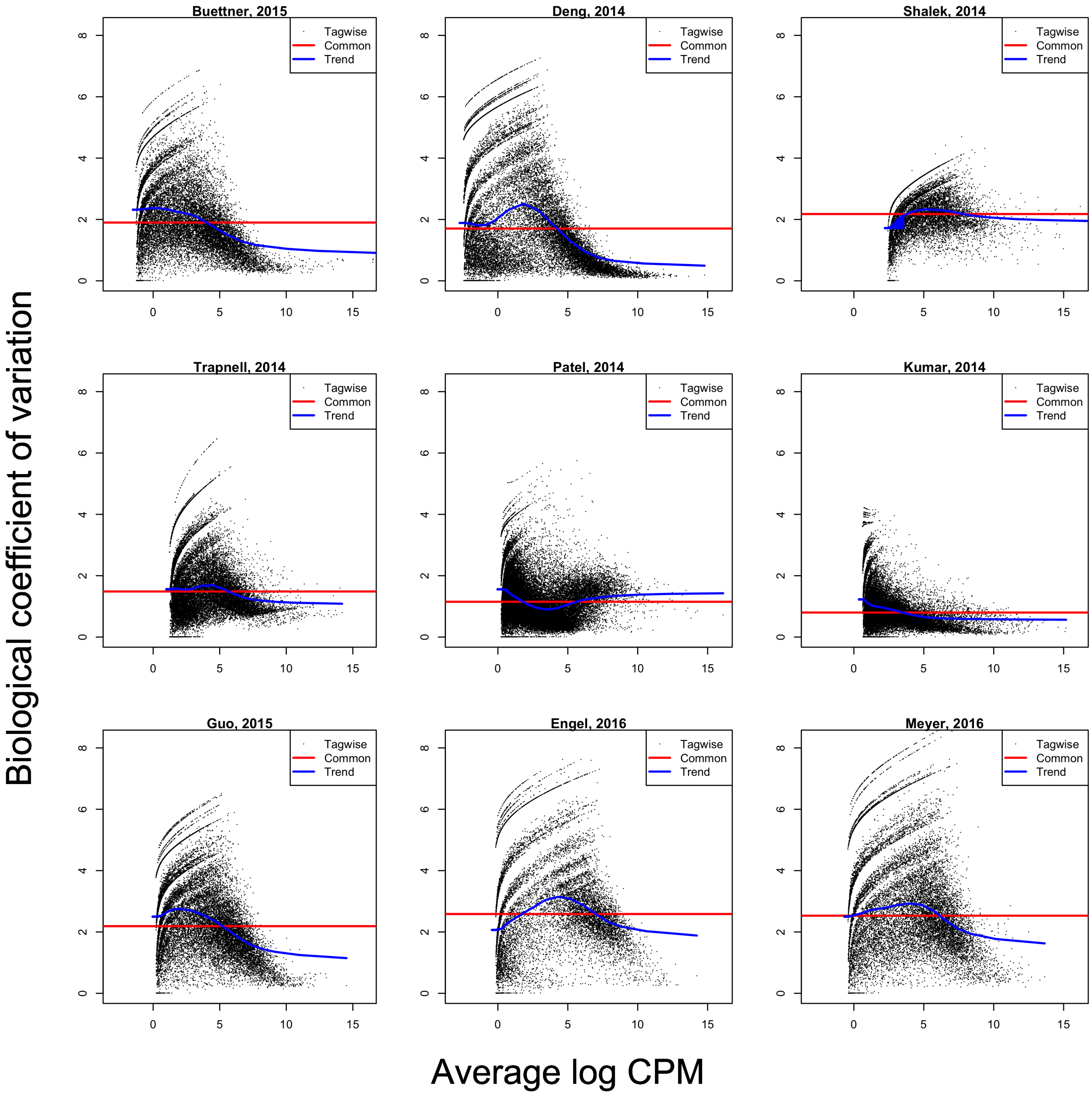

Figure S2: BCV plots for conquer datasets. Estimated biological coefficient of variation (BCV) vs. average log count per million (CPM), computed by `edgeR`, for conquer scRNA-seq datasets subsampled to $n = 10$ cells. The striped patterns reflect genes with many zero counts and high dispersion estimates, distorting the mean-variance relationship.

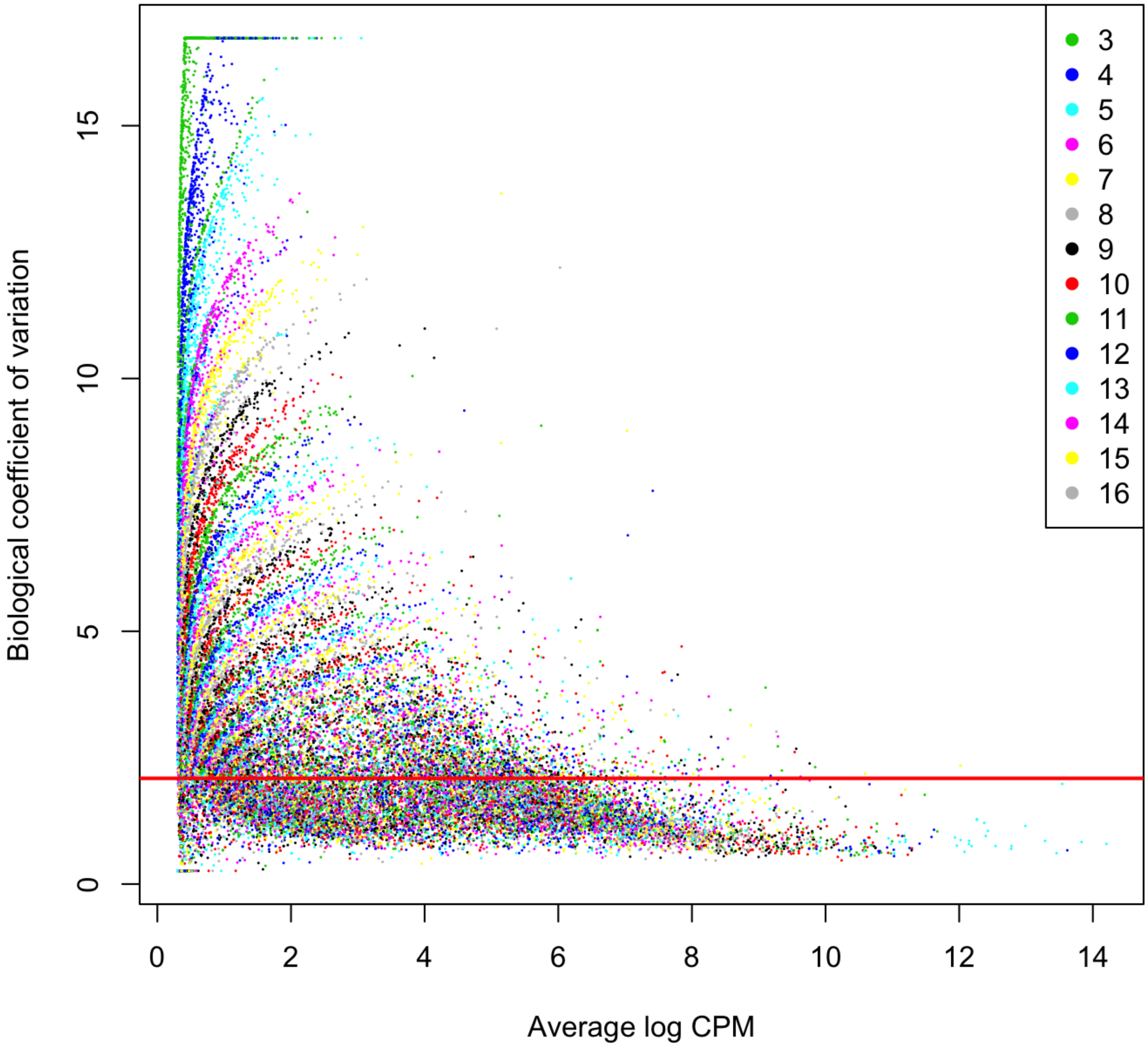

Figure S3: BCV plot for Trapnell dataset. Estimated biological coefficient of variation (BCV) vs. average log count per million (CPM), computed by `edgeR`, for the 72h subset of the Trapnell et al. [2013] scRNA-seq dataset, where colors represent the total number of positive counts across cells. The striped patterns originate from genes with few positive counts. They are also present in the lower half of the BCV plot, but can only be noticed with the coloring. The red line indicates the common dispersion estimated with `edgeR`.

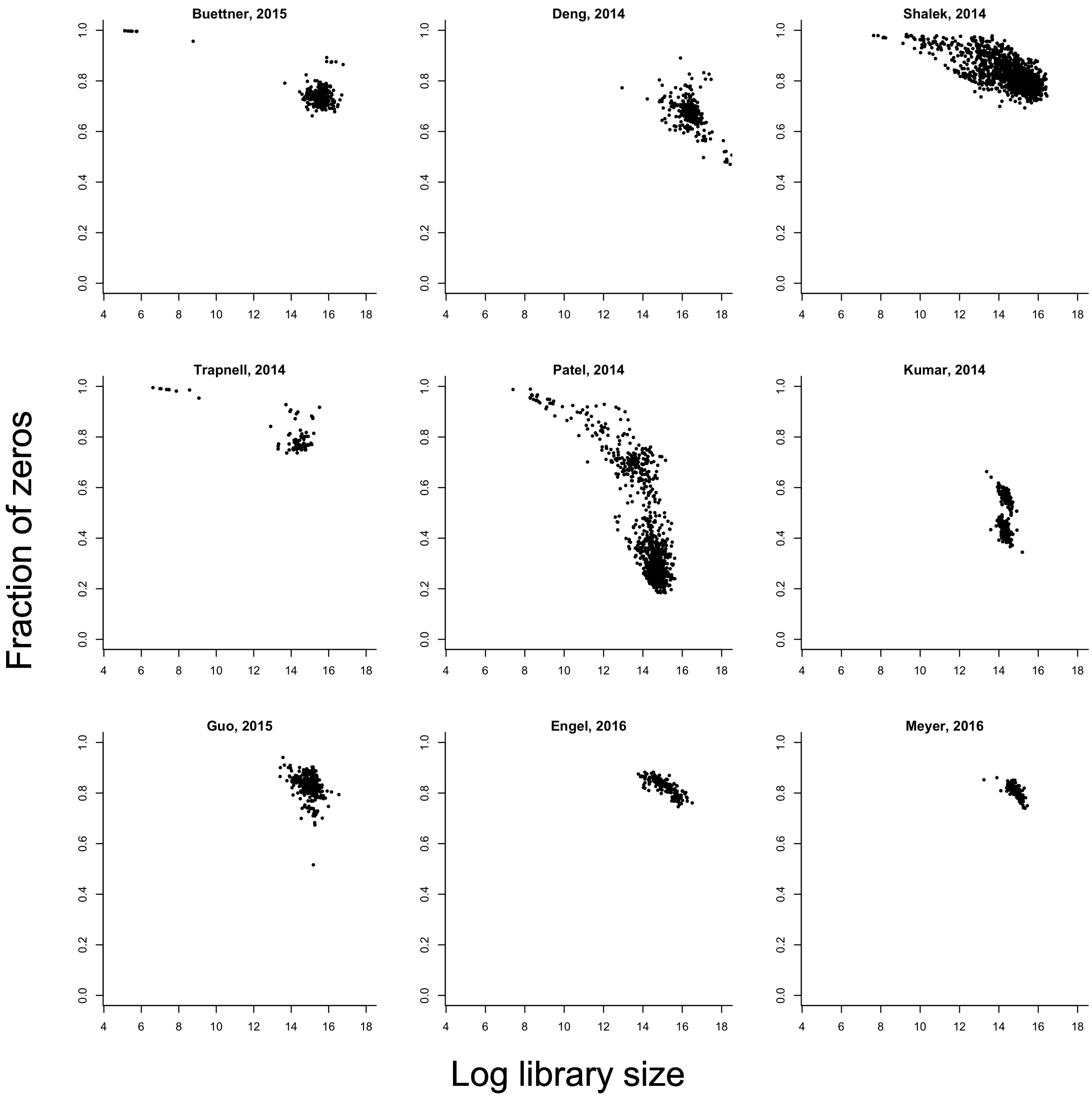

Figure S4: Zero proportion vs. log library size for conquer datasets. The fraction of zero counts for a cell is associated with library size, for scRNA-seq datasets downloaded from the conquer repository.

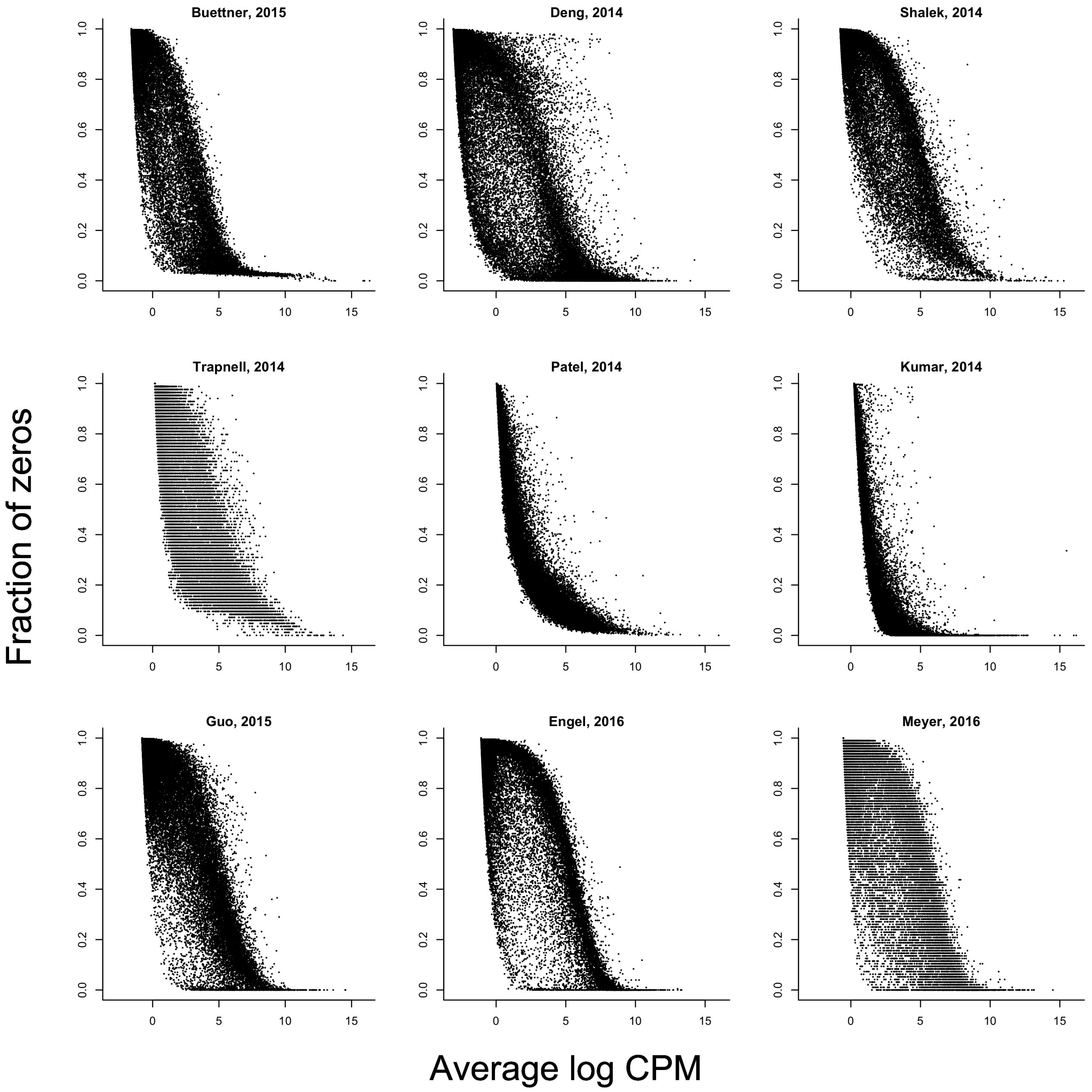

Figure S5: Zero proportion vs. average log CPM for conquer datasets. The fraction of zero counts for a gene is associated with its average expression, measured by the average log count per million (CPM), for scRNA-seq datasets downloaded from the conquer repository.

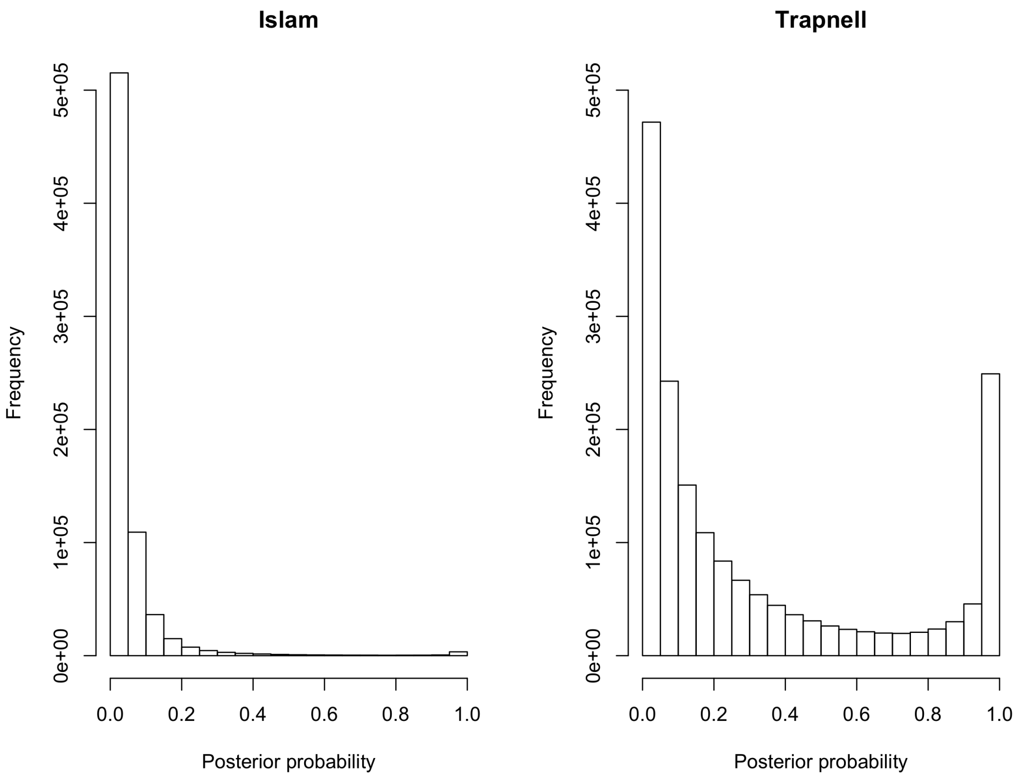

Figure S6: ZINB-WaVE posterior probabilities on real Islam and Trapnell scRNA-seq datasets. The histograms display the ZINB-WaVE estimated posterior probabilities of belonging to the negative binomial count component for all zeros in the Islam et al. [2011] and Trapnell et al. [2013] datasets. Many zeros are identified as excess zeros in the Islam dataset, while in the Trapnell dataset a reasonable proportion are estimated to be NB zeros.

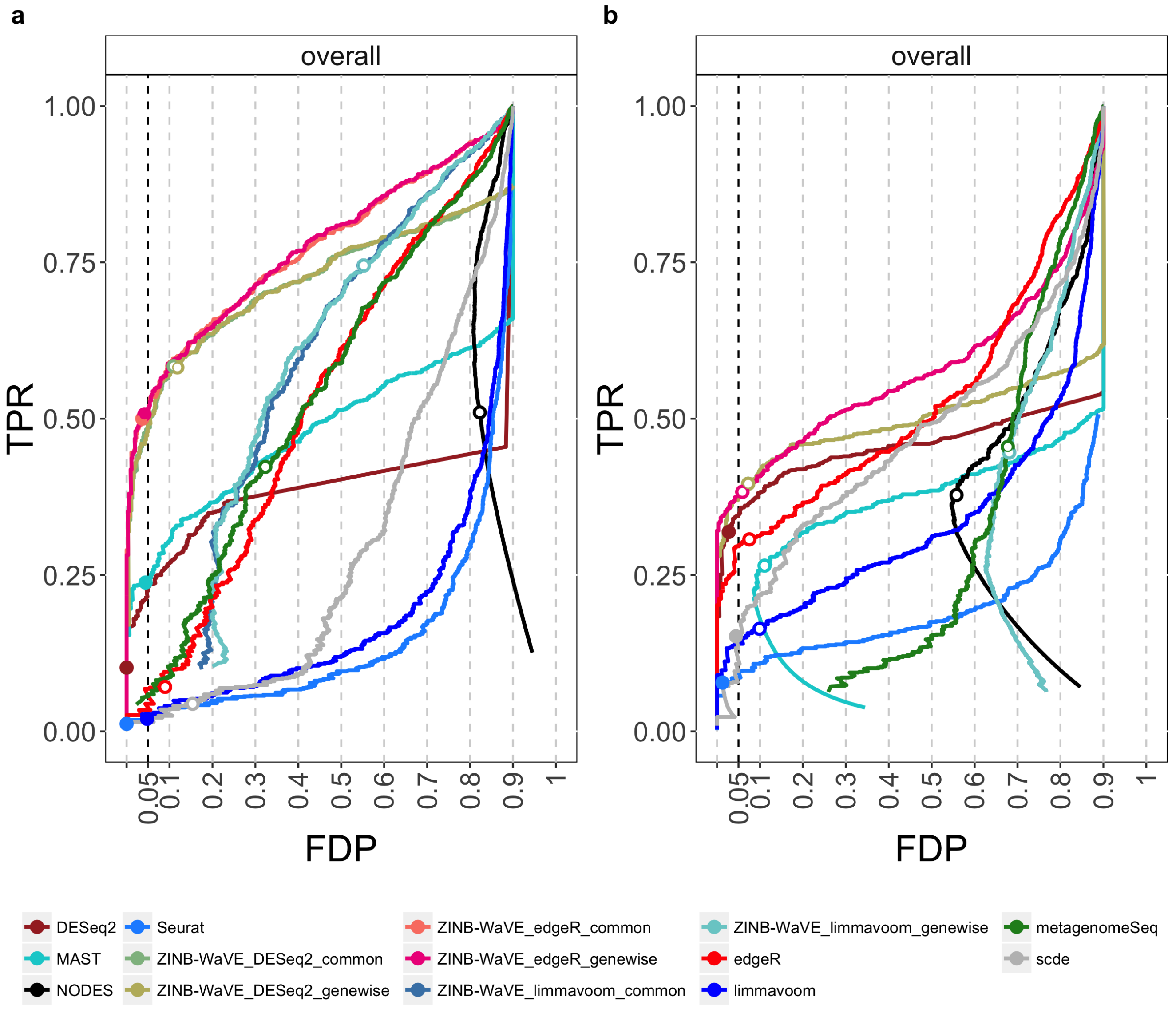

Figure S7: Comparison of DE methods on simulated scRNA-seq datasets. **(a)** scRNA-seq data simulated from Islam et al. [2011] dataset ($n = 90$). **(b)** scRNA-seq data simulated from Trapnell et al. [2013] dataset ($n = 150$). As in Figure 3, DE methods are compared based on scatterplots of the true positive rate (TPR) vs. the false discovery proportion (FDP). Circles represent working points on a nominal 5% FDR level and are filled if the empirical FDR (i.e., FDP) is below the nominal FDR. Methods based on ZINB-WaVE weights clearly outperform other methods for both simulated datasets. Note that the methods differ in performance between datasets, possibly because of a higher degree of zero inflation in the Islam dataset. The SCDE and metagenomeSeq methods, specifically developed to deal with excess zeros, are outperformed in both simulations by ZINB-WaVE-based methods and by DESeq2.

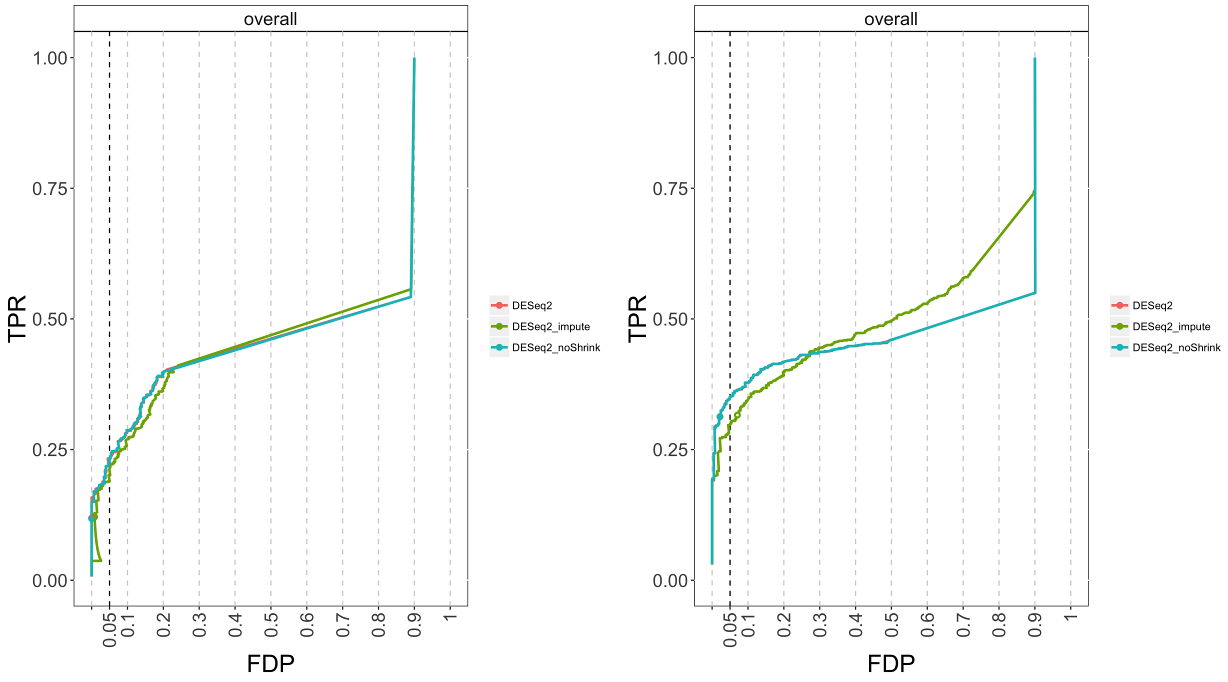

Figure S8: Comparison of DESeq2 variants on simulated scRNA-seq data. **(a)** scRNA-seq data simulated from Islam et al. [2011] dataset ($n = 90$). **(b)** scRNA-seq data simulated from Trapnell et al. [2013] dataset ($n = 150$). Three DESeq2 variants are compared based on scatterplots of the true positive rate (TPR) vs. the false discovery proportion (FDP). Circles represent working points on a nominal 5% FDR level and are filled if the empirical FDR (i.e., FDP) is below the nominal FDR. Enabling the imputation step in DESeq2 (method `DESeq2_impute`) results in a deterioration of performance on both datasets. Our default DESeq2 analysis (method `DESeq2`) has shrinkage of the fold-changes enabled, but disabling this option (method `DESeq2_noShrink`) does not seem to have a detrimental effect on performance. Note that the curve for `DESeq2_noShrink` is superimposed on the curve for `DESeq2` due to approximately identical performance.

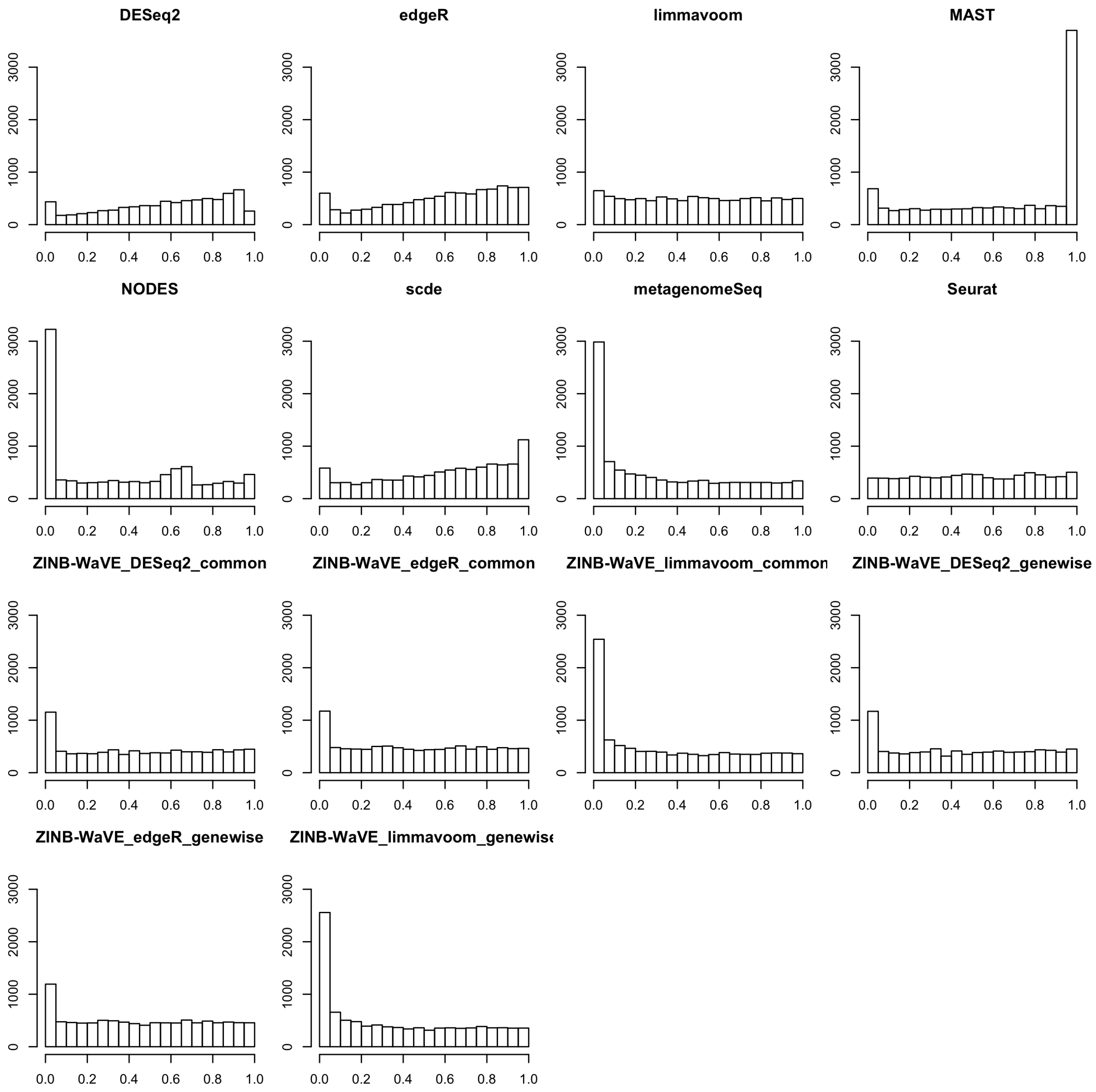

Figure S9: p-value distributions on simulated Islam scRNA-seq dataset ($n = 90$). The p-value distributions for methods based on ZINB-WaVE weights appear uniform for large p-values and enriched for low p-values, as would be expected from an appropriate statistical inference method on datasets with known effects. metagenomeSeq, NODES, and ZINB-WaVE-weighted limma-voom have anti-conservative p-value distributions, also evident in the FDP-TPR curves. SCDE and MAST have conservative p-value distributions.

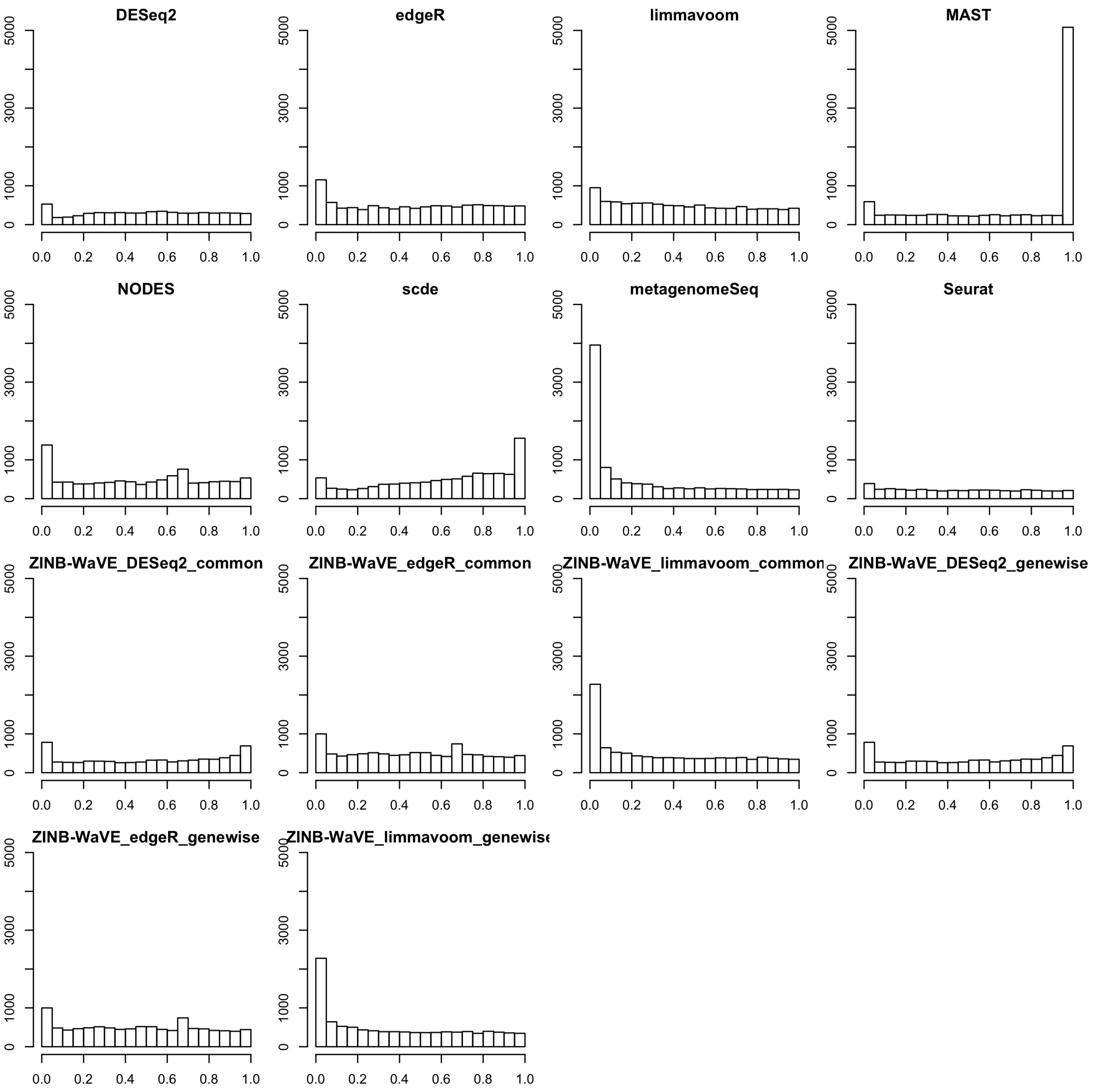

Figure S10: p-value distributions on simulated Trapnell scRNA-seq dataset ($n = 150$). The p-value distributions for methods based on ZINB-WaVE weights appear uniform for large p-values and enriched for low p-values, as would be expected from an appropriate statistical inference method on datasets with known effects. metagenomeSeq, NODES, and ZINB-WaVE-weighted limma-voom have anti-conservative p-value distributions, also evident in the FDP-TPR curves. SCDE and MAST have conservative p-value distributions.

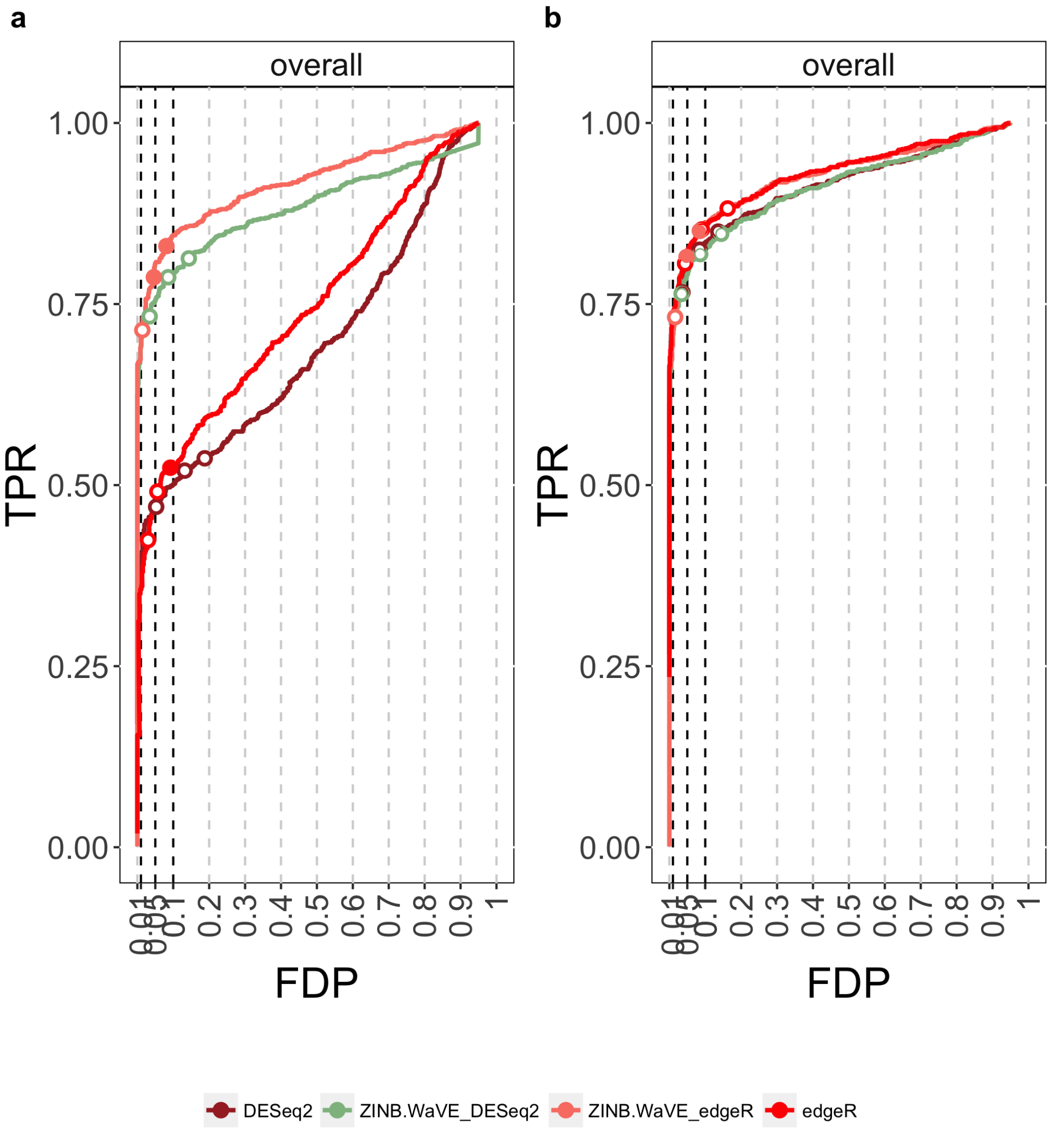

Figure S11: Comparison of edgeR and DESeq2 with and without ZINB-WaVE weights on simulated bulk RNA-seq datasets ($n = 10$). Bulk RNA-seq data were simulated from the Bottomly et al. [2011] dataset using the simulation framework of Zhou et al. [2014]. DE methods are compared based on scatterplots of the true positive rate (TPR) vs. the false discovery proportion (FDP). Circles represent working points on a nominal 5% FDR level and are filled if the empirical FDR (i.e., FDP) is below the nominal FDR. **(a)** Zero-inflated bulk RNA-seq dataset, where 5% of all counts were randomly replaced by zeros. Methods based on ZINB-WaVE weights correctly identify excess zeros, while standard unweighted bulk RNA-seq tools break down in performance due to overestimation of the dispersion parameters. **(b)** Bulk RNA-seq dataset. Methods based on ZINB-WaVE weights have a similar performance to their unweighted counterparts, highlighting that in the absence of zero inflation, it is not detrimental to use the ZINB-WaVE weights for differential expression analysis.

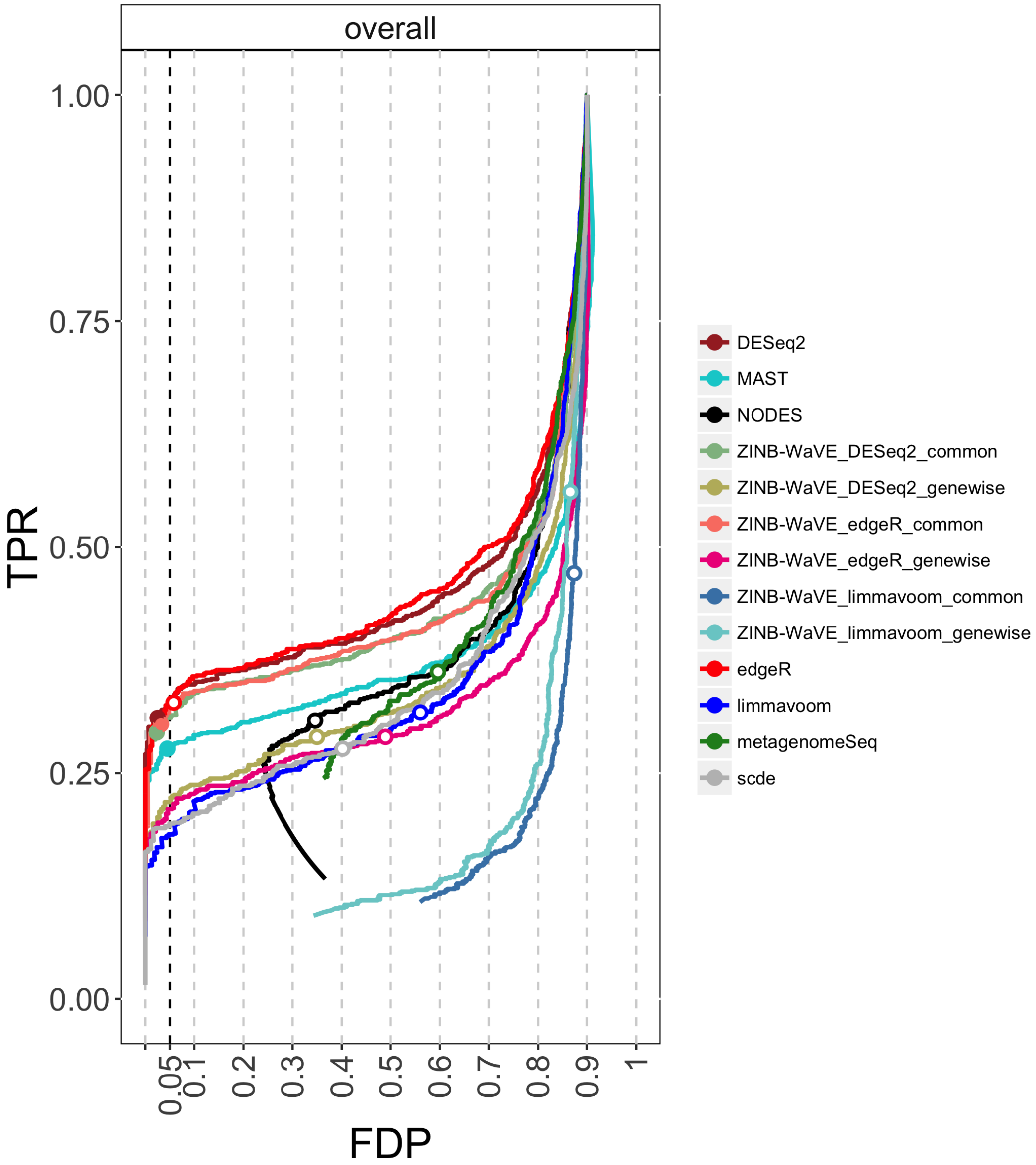

Figure S12: Comparison of DE methods on simulated scRNA-seq datasets. As in Figure 4, DE methods are compared based on FDP-TPR curves for data simulated from a 10x Genomics PBMC scRNA-seq dataset ($n = 1,200$). Circles represent working points on a nominal 5% FDR level and are filled if the empirical FDR (i.e., FDP) is below the nominal FDR. 10x Genomics sequencing typically involves high-throughput and massive multiplexing, resulting in very shallow sequencing depths and thus low counts, making it extremely difficult to identify excess zeros. Unweighted and ZINB-WaVE-weighted edgeR are tied for best performance, followed by ZINB-WaVE-weighted DESeq2. In general, bulk RNA-seq methods are performing well in this simulation, probably because the extremely high zero abundance in combination with low counts can be reasonably accommodated by the negative binomial distribution.

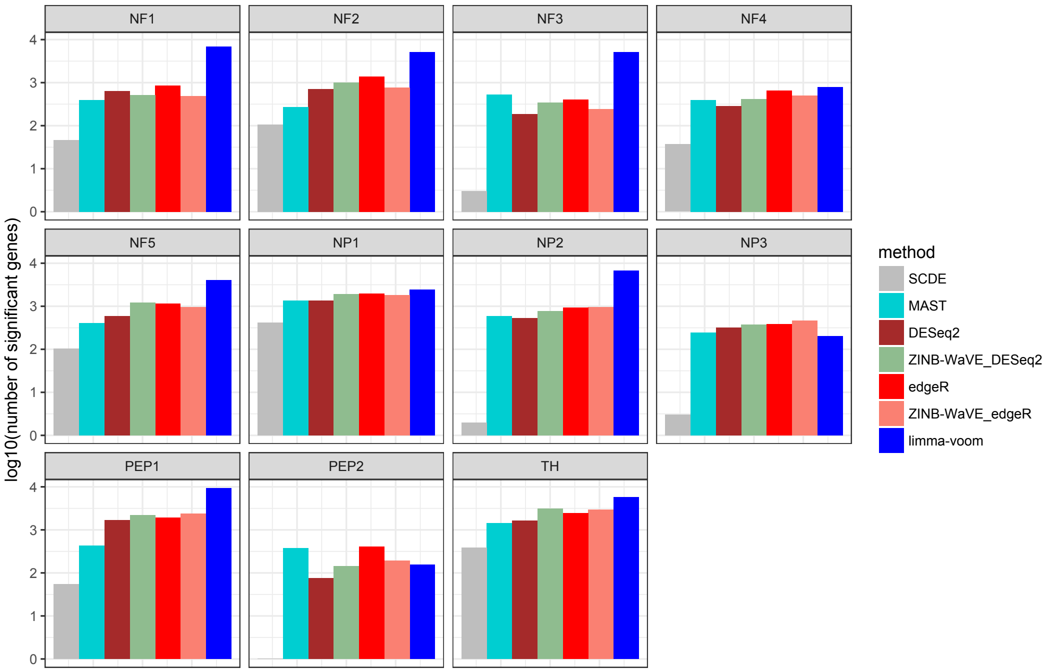

Figure S13: Differential expression results for Usoskin scRNA-seq dataset. The barplots provide the number of DE genes for the Usoskin et al. [2014] dataset, based on 7 DE methods comparing each cell type (panels) to all other cell types combined. The results for SCDE were obtained by assessing the number of genes with $|Z_{adj}| \geq 1.96$ (see supporting information for the original manuscript; `http://pklab.med.harvard.edu/scde/sensory.html`). All other methods are evaluated on a 5% nominal FDR level.

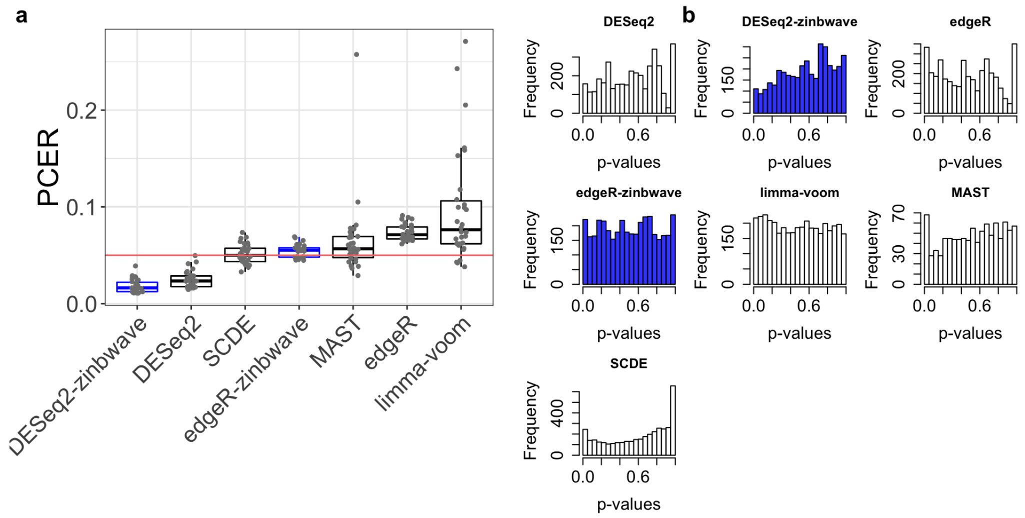

Figure S14: False positive control on mock null 10x Genomics PBMC datasets ($n = 2,700$ cells). **(a)** Boxplot of per-comparison error rate (PCER) for 30 mock null datasets for each of seven DE methods; ZINB-WaVE-weighted methods are highlighted in blue. **(b)** Histograms of unadjusted p-values for one of the datasets in (a). ZINB-WaVE was fit with intercept and mock cell type covariate in X, $V = \mathbf{1}_J$, $K = 0$ for W, common dispersion, and $\epsilon = 10^{12}$.

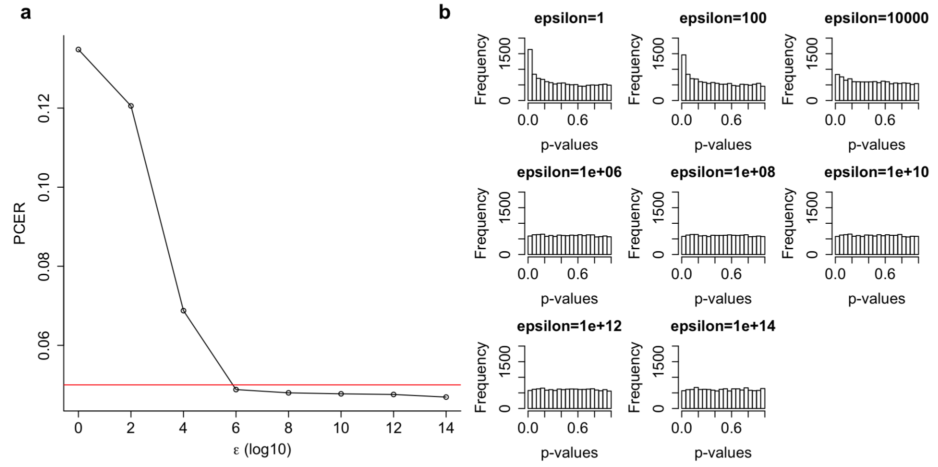

Figure S15: Impact of ZINB-WaVE regularization parameter for one mock null Usoskin dataset. **(a)** PCER as a function of the ZINB-WaVE regularization parameter ϵ. **(b)** Histograms of unadjusted p-values for different values of ϵ. DE genes for one mock null dataset are identified based on ZINB-WaVE-weighted edgeR, with an unadjusted p-value cut-off of 0.05. ZINB-WaVE was fit with intercept, mock cell type covariate, and batch covariate in X, $V = \mathbf{1}_J$, $K = 0$ for W, and common dispersion.

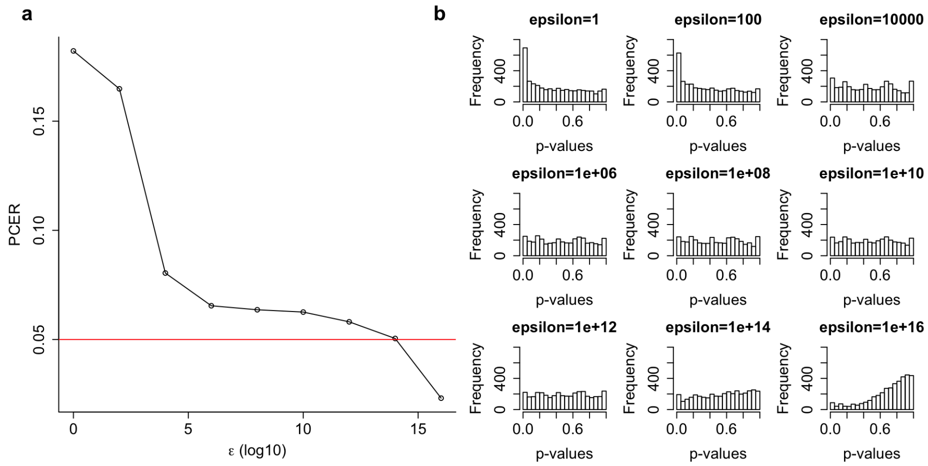

Figure S16: Impact of ZINB-WaVE regularization parameter for one mock null 10x Genomics PBMC dataset. **(a)** PCER as a function of the ZINB-WaVE regularization parameter ϵ. **(b)** Histograms of unadjusted p-values for different values of ϵ. DE genes for one mock null dataset are identified based on ZINB-WaVE-weighted edgeR, with an unadjusted p-value cut-off of 0.05. ZINB-WaVE was fit with intercept and mock cell type covariate in X, $V = \mathbf{1}_J$, $K = 0$ for W, and common dispersion.

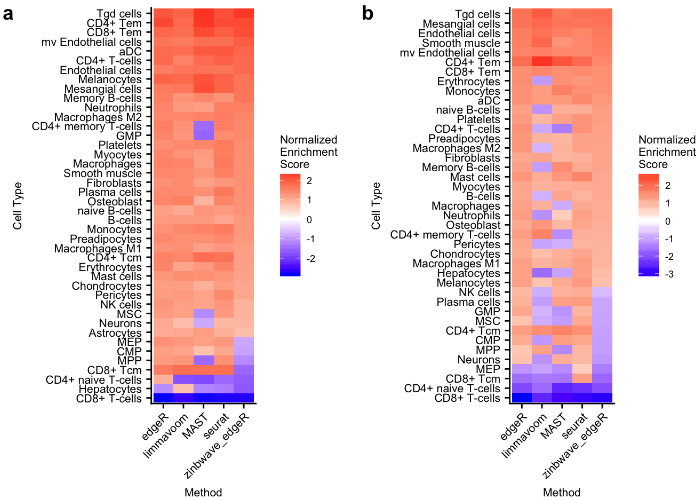

Figure S17: Gene set enrichment analysis based on PCA and ZINB-WaVE for 10x Genomics PBMC dataset. Pseudo-color images of normalized enrichment scores for gene set enrichment analysis, for differential expression between Seurat subclusters (CD4+ naive T-cells and CD4+ memory T-cells) based on **(a)** the first 10 principal components and **(b)** W from ZINB-WaVE with $K = 20$. ZINB-WaVE and GSEA parameters are as in Figure 6

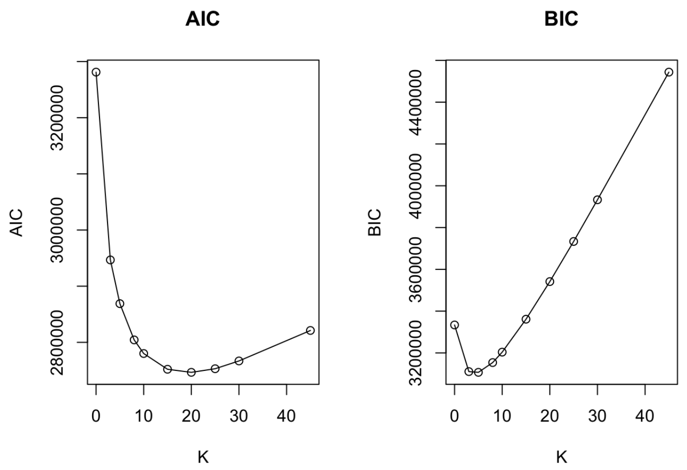

Figure S18: AIC and BIC for selecting the number of unknown cell-level covariates in ZINB-WaVE for 10x Genomics PBMC dataset. Panels show **(a)** the Akaike information criterion (AIC) and **(b)** the Bayesian information criterion (BIC) as a function of the number of unknown cell-level covariates K in the W matrix from the ZINB-WaVE model of Equations (3) and (4). ZINB-WaVE parameters are as in Figure 6.

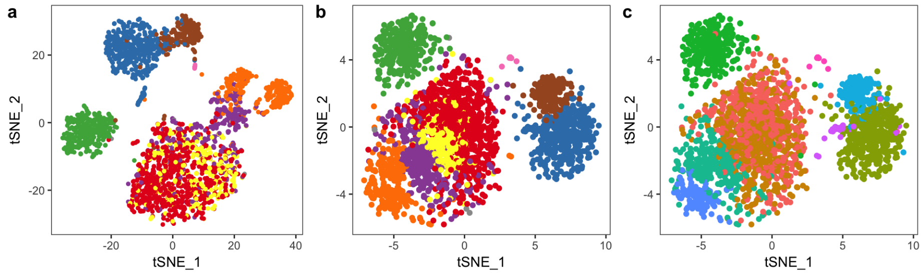

Figure S19: Clustering based on PCA and ZINB-WaVE for 10x Genomics PBMC dataset. **(a)** Scatterplot of first two t-SNE dimensions obtained from the first 10 principal components; cells are color-coded by Seurat graph-based clustering on ZINB-WaVE W ($K = 20$). **(b)** Scatterplot of first two t-SNE dimensions obtained from ZINB-WaVE W with $K = 20$; cells are color-coded by Seurat graph-based clustering on ZINB-WaVE W ($K = 20$). **(c)**. Scatterplot of first two t-SNE dimensions obtained from ZINB-WaVE W with $K = 20$; cells are color-coded by Seurat graph-based clustering on the first 10 principal components. In panels (a) and (b), CD4+ naive T-cells and CD4+ memory T-cells are, respectively, in yellow and red. Colors in panel (c) are the same as in Figure 6, where CD4+ naive T-cells and CD4+ memory T-cells are, respectively, in gold and red. ZINB-WaVE parameters are as in Figure 6.

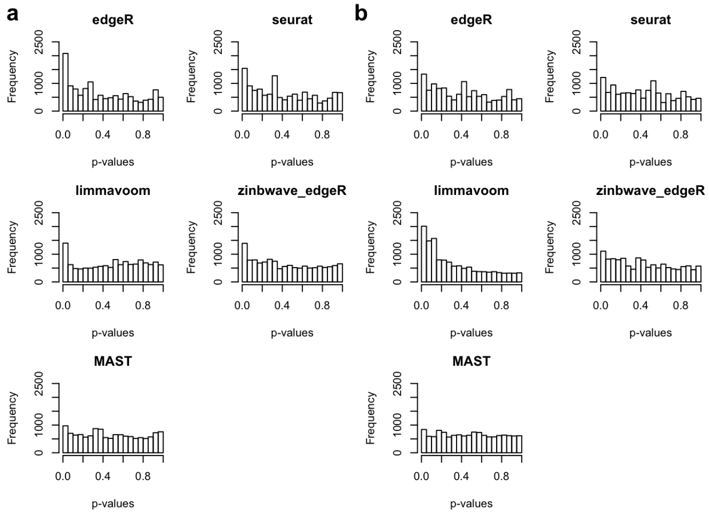

Figure S20: Differential expression between clusters based on PCA and ZINB-WaVE for 10x Genomics PBMC dataset. Histograms of unadjusted p-values from five methods for differential expression between Seurat subclusters (CD4+ naive T-cells and CD4+ memory T-cells) based on **(a)** the first 10 principal components and **(b)** W from ZINB-WaVE with $K = 20$. ZINB-WaVE parameters are as in Figure 6.

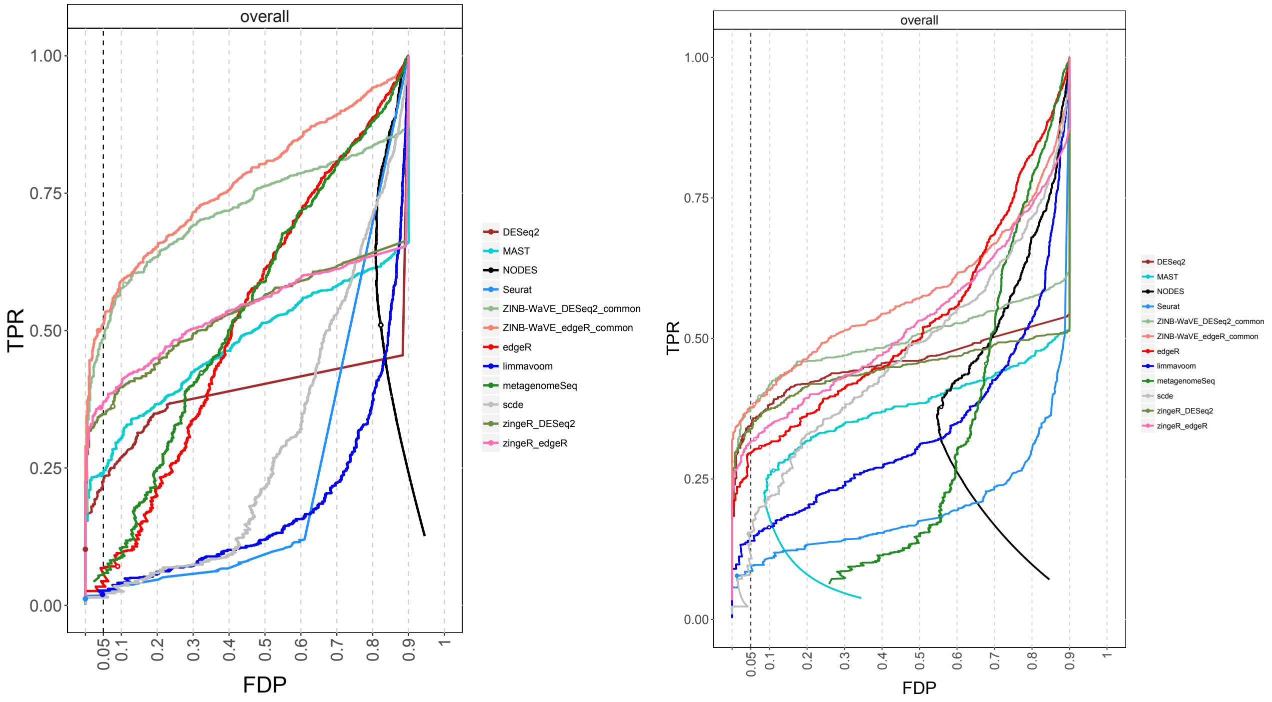

Figure S21: Comparison of DE methods on simulated scRNA-seq data, including the zingeR methods. Left panel: scRNA-seq data simulated from Islam et al. [2011] dataset ($n = 80$). Right panel: scRNA-seq data simulated from Trapnell et al. [2013] dataset ($n = 150$). DE methods are compared based on scatter plots of the true positive rate (TPR) vs. the false discovery proportion (FDP). Circles represent working points on a nominal 5% FDR level and are filled if the empirical FDR (i.e., FDP) is below the nominal FDR. The zingeR methods outperform all competing methods, except for the ZINB-WaVE methods. The DESeq2 curve in the left panel is cut off due to NA p-values resulting from independent filtering. The behavior in the lower half of the curve for MAST in the right panel is due to an extrapolation between two low working points.

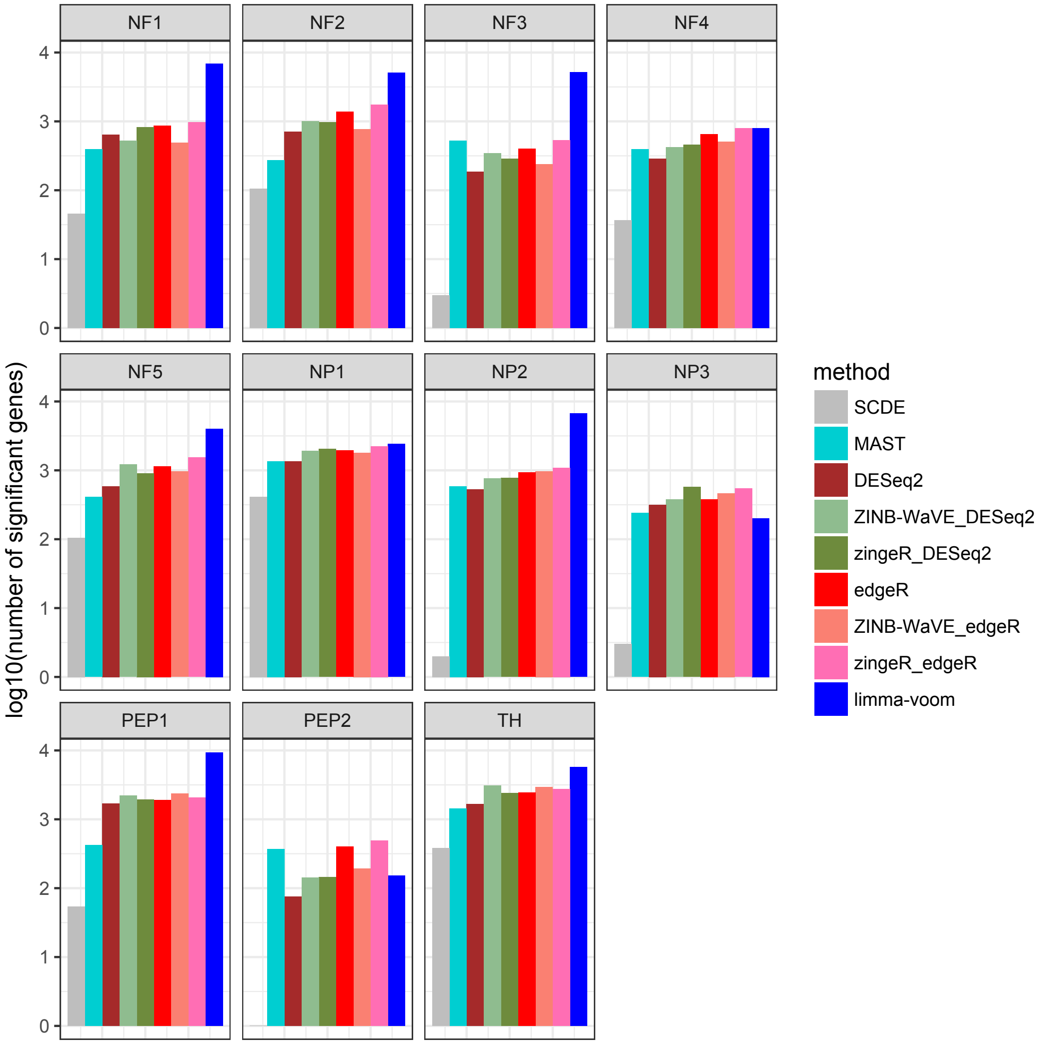

Figure S22: <u>Differential expression results for Usoskin scRNA-seq dataset.</u> The barplots provide the number of DE genes for the Usoskin et al. [2014] dataset, based on 9 DE methods comparing each cell type (panels) to all other cell types combined. The `zingeR` methods consistently have a higher number of DE genes as compared to their unweighted counterparts. The results for SCDE were obtained by assessing the number of genes with $|Z_{adj}| \geq 1.96$ (see supporting information for the original manuscript; `http://pklab.med.harvard.edu/scde/sensory.html`). All other methods are evaluated on a 5% nominal FDR level.

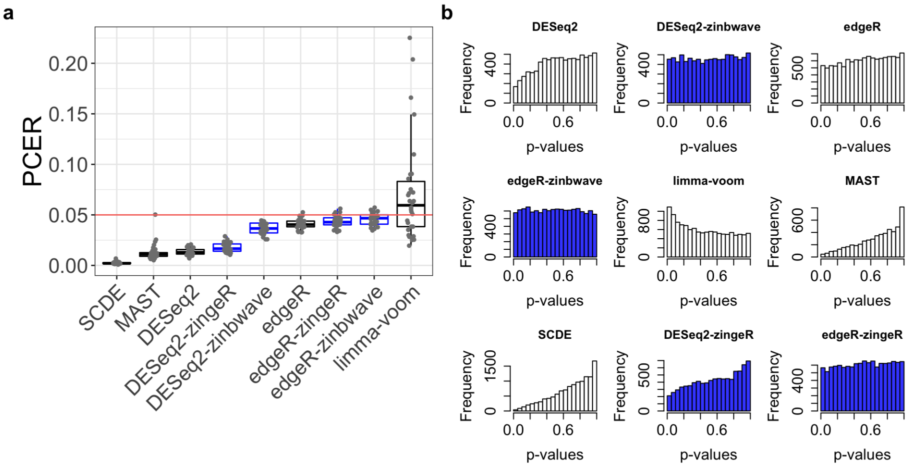

Figure S23: False positive control on mock null Usoskin datasets ($n = 622$ cells). **(a)** Boxplot of per-comparison error rate (PCER) for 30 mock null datasets for each of seven DE methods; ZINB-WaVE-weighted and zingeR-weighted methods are highlighted in blue. **(b)** Histogram of unadjusted p-values for one of the datasets in (a). ZINB-WaVE was fit with intercept, cell type covariate (actual or mock), and batch covariate (unless specified otherwise) in X, $V = \mathbf{1}_J$, $K = 0$ for W, common dispersion, and $\epsilon = 10^{12}$.

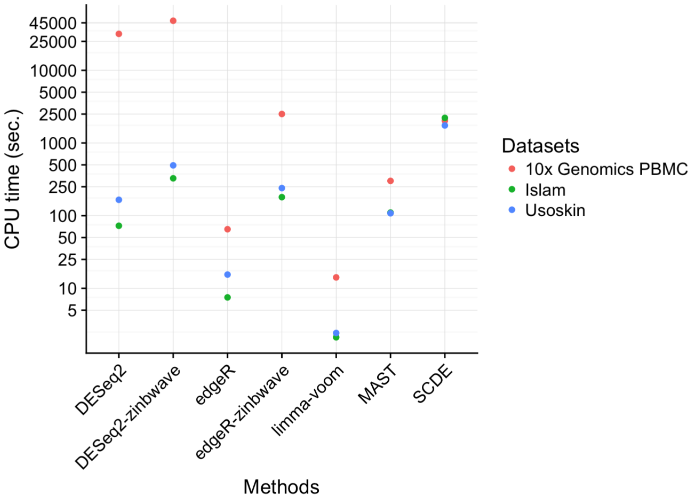

Figure S24: CPU time for different DE methods and scRNA-seq datasets. Mean CPU time (in seconds and on the log scale) for seven DE methods applied to three scRNA-seq datasets. For each method, the same parameters as in section "False positive rate control" were used. Colors correspond to different datasets. Islam dataset: Actual read counts for $n = 92$ cells. Usoskin dataset: Read counts for $n = 90$ cells from one of the mock null datasets used in Figure 5; batch was included as a covariate for all methods. 10x Genomics PBMC dataset: Only the $n = 1,151$ cells in the CD4+ T-cells clusters were used. $10,000$ genes were sampled at random for each dataset. Computations were done on a MacBook Pro with four 2.7 GHz Intel Core i5 CPUs and 8 GB of RAM. Although some methods allow the use of multiple cores, only one core was used here for comparison purposes. ZINB-WaVE was fit with intercept and cell type covariate (and batch covariate for Usoskin dataset) in X, $V = \mathbf{1}_J$, $K = 0$ for W, common dispersion, and $\epsilon = 10^{12}$.

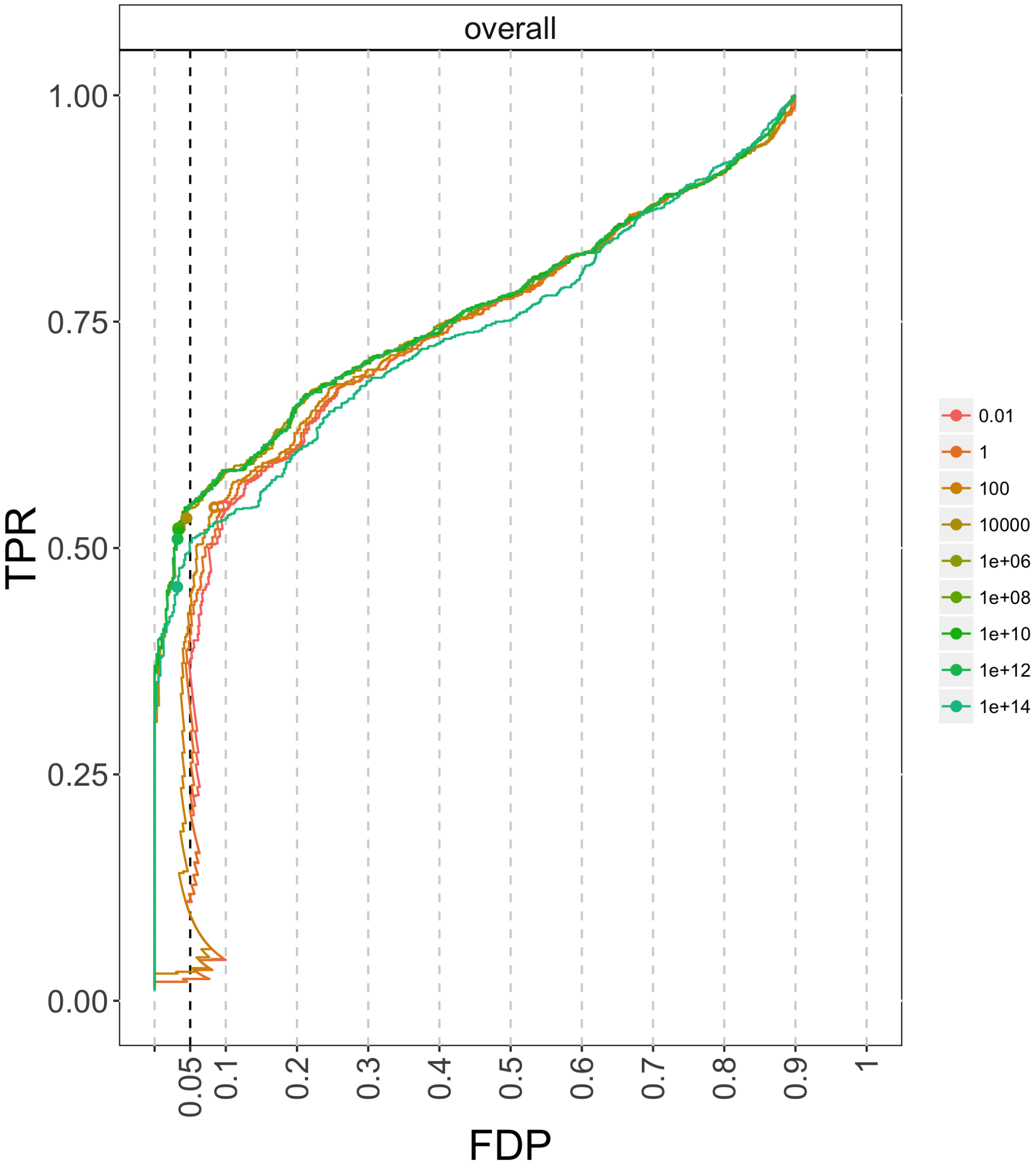

Figure S25: Impact of ZINB-WaVE regularization parameter on ZINB-WaVE-weighted edgeR for simulated Islam dataset. The different FDP-TPR curves correspond to ZINB-WaVE-weighted edgeR analyses with varying values for the ZINB-WaVE regularization parameter ϵ, as specified in the legend. The regularization parameter ϵ has a big influence on performance due to the different degrees of shrinkage applied to the ZINB-WaVE parameter estimates. We have found that setting $\epsilon = 10^{12}$ works well in general, but further research is needed to select optimal values of the penalty parameter.

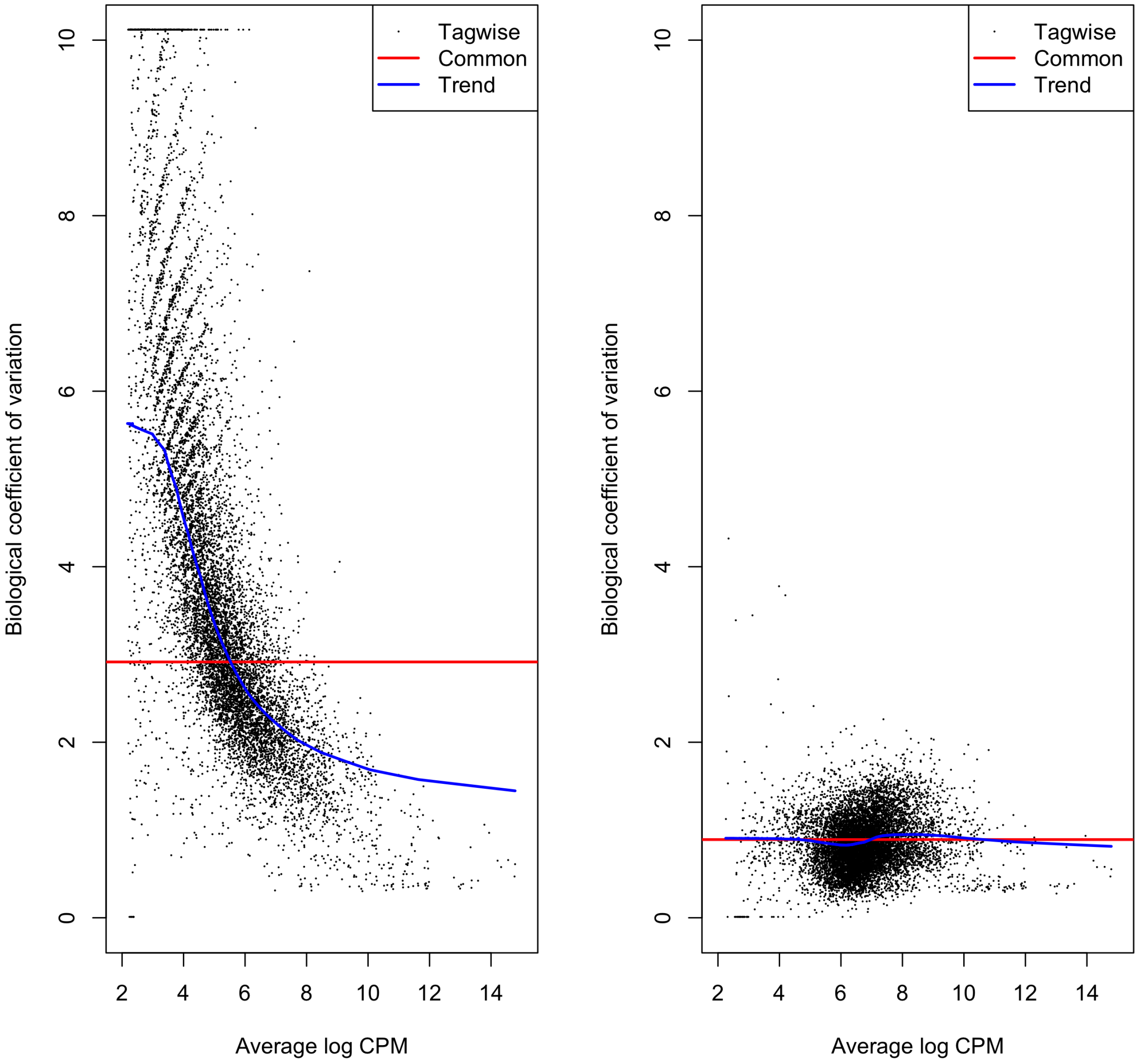

Figure S26: Effect of weighting on the mean-variance relationship for the simulated Islam scRNA-seq dataset. Estimated biological coefficient of variation (BCV) vs. average log count per million (CPM), computed by `edgeR`. Left panel: The BCV plot based on unweighted observations shows a strong mean-variance relationship, with very high dispersion estimates for lowly-expressed genes. Right panel: The BCV plot on the same dataset, where excess zeros identified by ZINB-WaVE are downweighted, shows much lower dispersion estimates.

References

Saiful Islam, Una Kjällquist, Annalena Moliner, Pawel Zajac, Jian-Bing Fan, Peter Lönnerberg, and Sten Linnarsson. Characterization of the single-cell transcriptional landscape by highly multiplex RNA-seq. Genome research, 21 (7):1160–7, jul 2011. ISSN 1549-5469. doi: 10.1101/gr.110882.110. URL http://www.pubmedcentral.nih.gov/articlerender.fcgi?artid=3129258{&}tool=pmcentrez{&}rendertype=abstract.

Joseph K Pickrell, John C Marioni, Athma A Pai, Jacob F Degner, Barbara E Engelhardt, Everlyne Nkadori, Jean-Baptiste Veyrieras, Matthew Stephens, Yoav Gilad, and Jonathan K Pritchard. Understanding mechanisms underlying human gene expression variation with RNA sequencing. Nature, 464(7289):768–72, apr 2010. ISSN 1476-4687. doi: 10.1038/nature08872. URL http://www.ncbi.nlm.nih.gov/pubmed/20220758http://www.pubmedcentral.nih.gov/articlerender.fcgi?artid=PMC3089435.

Cole Trapnell, David G Hendrickson, Martin Sauvageau, Loyal Goff, John L Rinn, and Lior Pachter. Differential analysis of gene regulation at transcript resolution with RNA-seq. Nature biotechnology, 31(1):46–53, jan 2013. ISSN 1546-1696. doi: 10.1038/nbt.2450. URL http://dx.doi.org/10.1038/nbt.2450.

Daniel Bottomly, Nicole A R Walter, Jessica Ezzell Hunter, Priscila Darakjian, Sunita Kawane, Kari J Buck, Robert P Searles, Michael Mooney, Shannon K McWeeney, and Robert Hitzemann. Evaluating gene expression in C57BL/6J and DBA/2J mouse striatum using RNA-Seq and microarrays. PloS one, 6(3):e17820, jan 2011. ISSN 1932-6203. doi: 10.1371/journal.pone.0017820. URL http://www.pubmedcentral.nih.gov/articlerender.fcgi?artid=3063777{&}tool=pmcentrez{&}rendertype=abstract.

Xiaobei Zhou, Helen Lindsay, and Mark D Robinson. Robustly detecting differential expression in RNA sequencing data using observation weights. Nucleic acids research, 42(11):e91, jun 2014. ISSN 1362-4962. doi: 10.1093/nar/gku310. URL http://www.ncbi.nlm.nih.gov/pubmed/24753412http://www.pubmedcentral.nih.gov/articlerender.fcgi?artid=PMC4066750.

Dmitry Usoskin, Alessandro Furlan, Saiful Islam, Hind Abdo, Peter Lönnerberg, Daohua Lou, Jens Hjerling-Leffler, Jesper Haeggström, Olga Kharchenko, Peter V Kharchenko, Sten Linnarsson, and Patrik Ernfors. Unbiased classification of sensory neuron types by large-scale single-cell RNA sequencing. Nature Neuroscience, 18(1):145–153, nov 2014. ISSN 1097-6256. doi: 10.1038/nn.3881. URL http://www.nature.com/doifinder/10.1038/nn.3881.
