# Supplementary material for: Observation weights unlock bulk RNA-seq tools for zero inflation and single-cell applications
Source: Genome Biol. 2018 Feb 26;19:24. doi: 10.1186/s13059-018-1406-4 (PMC6251479; doi:10.1186/s13059-018-1406-4)
Supplement: Supplementary file 4 — countsimQC evaluation of simulated 10x dataset. (HTML 9933 kb) [file 13059_2018_1406_MOESM4_ESM.html]

Comparison of characteristic features across count data sets


Code 

- Show All Code
- Hide All Code

# Comparison of characteristic features across count data sets

## Description

evaluation of simulated 10X PBMC dataset

#### Design formulae

The following model formulae were used in the dispersion calculations for the different data sets. Note that if a count matrix or data frame was provided in place of a *DESeqDataSet* for some data set, the corresponding design formula is set to `~1`, thus assuming that all samples are to be treated as replicates.

```
original :  ~ celltype 
zingeR :  ~ grp
```

## Data set dimensions

These bar plots show the number of samples (columns) and variables (rows) in each data set.

### Number of samples (columns)

### Number of variables (rows)

## Dispersion/BCV plots

Disperson/BCV plots show the association between the average abundance and the dispersion or “biological coefficient of variation” (sqrt(dispersion)), as calculated by `edgeR` (Robinson, McCarthy, and Smyth 2010) and `DESeq2` (Love, Huber, and Anders 2014). In the `edgeR` plot, the estimate of the prior degrees of freedom is indicated.

### edgeR

The black dots represent the tagwise dispersion estimates, the red line the common dispersion and the blue curve represents the trended dispersion estimates. For further information about the dispersion estimation in `edgeR`, see Chen, Lun, and Smyth (2014).

### Pairwise comparisons - edgeR

No statistics were calculated, since the ‘calculateStatistics’ argument to ‘countsimQCReport()’ was set to FALSE. To perform pairwise quantitative comparisons between data sets, set this argument to TRUE. Note, however, that this increases the runtime significantly.

### DESeq2

The black dots are the gene-wise dispersion estimates, the red curve the fitted mean-dispersion relationship and the blue circles represent the final dispersion estimates.For further information about the dispersion estimation in `DESeq2`, see Love, Huber, and Anders (2014).

### Pairwise comparisons - DESeq2

No statistics were calculated, since the ‘calculateStatistics’ argument to ‘countsimQCReport()’ was set to FALSE. To perform pairwise quantitative comparisons between data sets, set this argument to TRUE. Note, however, that this increases the runtime significantly.

## Mean-variance plots

These scatter plots show the relation between the empirical mean and variance of the variables. The difference between these mean-variance plots and the mean-dispersion plots above is that the plots in this section do not take the information about the experimental design and sample grouping into account, but simply display the mean and variance of log2(CPM) estimates across all samples, calculated using the `cpm` function from `edgeR` (Robinson, McCarthy, and Smyth 2010), with a prior count of 2.

### Separate scatter plots

### Overlaid scatter plots

### Pairwise comparisons

No statistics were calculated, since the ‘calculateStatistics’ argument to ‘countsimQCReport()’ was set to FALSE. To perform pairwise quantitative comparisons between data sets, set this argument to TRUE. Note, however, that this increases the runtime significantly.

## Library sizes

These plots illustrate the distribution of the total read count per sample, i.e., the column sums of the respective data matrices.

### Separate histograms

### Overlaid histograms

### Density plots

### Density plots (filled)

### Box plots

### Violin plots

### Empirical cumulative distribution function

### Pairwise comparisons

No statistics were calculated, since the ‘calculateStatistics’ argument to ‘countsimQCReport()’ was set to FALSE. To perform pairwise quantitative comparisons between data sets, set this argument to TRUE. Note, however, that this increases the runtime significantly.

## TMM normalization factors

The plots below show the distribution of the TMM normalization factors (Robinson and Oshlack 2010), intended to adjust for differences in RNA composition, as calculated by `edgeR` (Robinson, McCarthy, and Smyth 2010).

### Separate histograms

### Overlaid histograms

### Density plots

### Density plots (filled)

### Box plots

### Violin plots

### Empirical cumulative distribution function

### Pairwise comparisons

No statistics were calculated, since the ‘calculateStatistics’ argument to ‘countsimQCReport()’ was set to FALSE. To perform pairwise quantitative comparisons between data sets, set this argument to TRUE. Note, however, that this increases the runtime significantly.

## Effective library sizes

These plots show the distribution of the “effective library sizes”, defined as the total count per sample multiplied by the corresponding TMM normalization factor.

### Separate histograms

### Overlaid histograms

### Density plots

### Density plots (filled)

### Box plots

### Violin plots

### Empirical cumulative distribution function

### Pairwise comparisons

No statistics were calculated, since the ‘calculateStatistics’ argument to ‘countsimQCReport()’ was set to FALSE. To perform pairwise quantitative comparisons between data sets, set this argument to TRUE. Note, however, that this increases the runtime significantly.

## Expression distributions (average log CPM)

The plots in this section show the distribution of average abundance values for the variables. The abundances are log CPM values calculated by `edgeR`.

### Separate histograms

### Overlaid histograms

### Density plots

### Density plots (filled)

### Box plots

### Violin plots

### Empirical cumulative distribution function

### Pairwise comparisons

No statistics were calculated, since the ‘calculateStatistics’ argument to ‘countsimQCReport()’ was set to FALSE. To perform pairwise quantitative comparisons between data sets, set this argument to TRUE. Note, however, that this increases the runtime significantly.

## Fraction zeros per sample

These plots show the distribution of the fraction of zeros observed per sample (column) in the count matrices.

### Separate histograms

### Overlaid histograms

### Density plots

### Density plots (filled)

### Box plots

### Violin plots

### Empirical cumulative distribution function

### Pairwise comparisons

No statistics were calculated, since the ‘calculateStatistics’ argument to ‘countsimQCReport()’ was set to FALSE. To perform pairwise quantitative comparisons between data sets, set this argument to TRUE. Note, however, that this increases the runtime significantly.

## Fraction zeros per feature

These plots illustrate the distribution of the fraction of zeros observed per variable (row) in the count matrices.

### Separate histograms

### Overlaid histograms

### Density plots

### Density plots (filled)

### Box plots

### Violin plots

### Empirical cumulative distribution function

### Pairwise comparisons

No statistics were calculated, since the ‘calculateStatistics’ argument to ‘countsimQCReport()’ was set to FALSE. To perform pairwise quantitative comparisons between data sets, set this argument to TRUE. Note, however, that this increases the runtime significantly.

## Sample-sample correlations

The plots below show the distribution of Spearman correlation coefficients for pairs of samples, calculated from the log(CPM) values obtained via the `cpm` function from `edgeR`, with a prior.count of 2. If there are more than `r maxNForCorr` samples in a data set, the pairwise correlations between `r maxNForCorr` randomly selected samples are shown.

### Separate histograms

### Overlaid histograms

### Density plots

### Density plots (filled)

### Box plots

### Violin plots

### Empirical cumulative distribution function

### Pairwise comparisons

No statistics were calculated, since the ‘calculateStatistics’ argument to ‘countsimQCReport()’ was set to FALSE. To perform pairwise quantitative comparisons between data sets, set this argument to TRUE. Note, however, that this increases the runtime significantly.

## Variable-variable correlations

These plots illustrate the distribution of Spearman correlation coefficients for pairs of variables, calculated from the log(CPM) values obtained via the `cpm` function from `edgeR`, with a prior.count of 2. Only non-constant variables are considered, and if there are more than 500 such variables in a data set, the pairwise correlations between 500 randomly selected variables are shown.

### Separate histograms

### Overlaid histograms

### Density plots

### Density plots (filled)

### Box plots

### Violin plots

### Empirical cumulative distribution function

### Pairwise comparisons

No statistics were calculated, since the ‘calculateStatistics’ argument to ‘countsimQCReport()’ was set to FALSE. To perform pairwise quantitative comparisons between data sets, set this argument to TRUE. Note, however, that this increases the runtime significantly.

## Library size vs fraction zeros

These scatter plots show the association between the total count (column sums) and the fraction of zeros observed per sample.

### Separate scatter plots

### Overlaid scatter plots

### Pairwise comparisons

No statistics were calculated, since the ‘calculateStatistics’ argument to ‘countsimQCReport()’ was set to FALSE. To perform pairwise quantitative comparisons between data sets, set this argument to TRUE. Note, however, that this increases the runtime significantly.

## Mean expression vs fraction zeros

These scatter plots show the association between the average abundance and the fraction of zeros observed per variable. The abundance is defined as the log(CPM) values as calculated by `edgeR`.

### Separate scatter plots

### Overlaid scatter plots

### Pairwise comparisons

No statistics were calculated, since the ‘calculateStatistics’ argument to ‘countsimQCReport()’ was set to FALSE. To perform pairwise quantitative comparisons between data sets, set this argument to TRUE. Note, however, that this increases the runtime significantly.

## Session info

```
## R version 3.4.1 (2017-06-30)
## Platform: x86_64-apple-darwin15.6.0 (64-bit)
## Running under: OS X El Capitan 10.11.6
## 
## Matrix products: default
## BLAS: /System/Library/Frameworks/Accelerate.framework/Versions/A/Frameworks/vecLib.framework/Versions/A/libBLAS.dylib
## LAPACK: /Library/Frameworks/R.framework/Versions/3.4/Resources/lib/libRlapack.dylib
## 
## locale:
## [1] en_US.UTF-8/en_US.UTF-8/en_US.UTF-8/C/en_US.UTF-8/en_US.UTF-8
## 
## attached base packages:
## [1] parallel  stats4    stats     graphics  grDevices utils     datasets 
## [8] methods   base     
## 
## other attached packages:
##  [1] bindrcpp_0.2               cellrangerRkit_2.0.0      
##  [3] Rmisc_1.5                  plyr_1.8.4                
##  [5] lattice_0.20-35            bit64_0.9-7               
##  [7] bit_1.1-12                 Matrix_1.2-11             
##  [9] countsimQC_0.5.0           MultiAssayExperiment_1.2.1
## [11] mgcv_1.8-18                nlme_3.1-131              
## [13] cowplot_0.8.0              ggplot2_2.2.1             
## [15] knitr_1.17                 RColorBrewer_1.1-2        
## [17] MAST_1.2.1                 genefilter_1.58.1         
## [19] iCOBRA_1.4.0               DESeq2_1.17.13            
## [21] scales_0.5.0               edgeR_3.19.3              
## [23] limma_3.32.5               doParallel_1.0.10         
## [25] iterators_1.0.8            foreach_1.4.3             
## [27] BiocParallel_1.10.1        zinbwave_0.99.6           
## [29] SummarizedExperiment_1.6.3 DelayedArray_0.2.7        
## [31] matrixStats_0.52.2         Biobase_2.36.2            
## [33] GenomicRanges_1.28.4       GenomeInfoDb_1.12.2       
## [35] IRanges_2.10.2             S4Vectors_0.14.3          
## [37] BiocGenerics_0.22.0       
## 
## loaded via a namespace (and not attached):
##  [1] Rtsne_0.13              colorspace_1.3-2       
##  [3] rprojroot_1.2           htmlTable_1.9          
##  [5] XVector_0.16.0          base64enc_0.1-3        
##  [7] gsl_1.9-10.3            DT_0.2                 
##  [9] AnnotationDbi_1.38.2    mvtnorm_1.0-6          
## [11] codetools_0.2-15        splines_3.4.1          
## [13] zingeR_0.1.0            geneplotter_1.54.0     
## [15] Formula_1.2-2           annotate_1.54.0        
## [17] cluster_2.0.6           pheatmap_1.0.8         
## [19] shinydashboard_0.6.1    stabledist_0.7-1       
## [21] copula_0.999-17         shiny_1.0.5            
## [23] compiler_3.4.1          backports_1.1.0        
## [25] assertthat_0.2.0        lazyeval_0.2.0         
## [27] acepack_1.4.1           htmltools_0.3.6        
## [29] tools_3.4.1             gtable_0.2.0           
## [31] glue_1.1.1              GenomeInfoDbData_0.99.0
## [33] reshape2_1.4.2          dplyr_0.7.4            
## [35] Rcpp_0.12.13            softImpute_1.4         
## [37] gdata_2.18.0            stringr_1.2.0          
## [39] irlba_2.2.1             mime_0.5               
## [41] gtools_3.5.0            XML_3.98-1.9           
## [43] zlibbioc_1.22.0         randtests_1.0          
## [45] shinyBS_0.61            rhdf5_2.20.0           
## [47] yaml_2.1.14             memoise_1.1.0          
## [49] gridExtra_2.3           UpSetR_1.3.3           
## [51] rpart_4.1-11            latticeExtra_0.6-28    
## [53] stringi_1.1.5           RSQLite_2.0            
## [55] pcaPP_1.9-72            checkmate_1.8.3        
## [57] caTools_1.17.1          rlang_0.1.2            
## [59] pkgconfig_2.0.1         bitops_1.0-6           
## [61] evaluate_0.10.1         purrr_0.2.3            
## [63] ROCR_1.0-7              bindr_0.1              
## [65] labeling_0.3            htmlwidgets_0.9        
## [67] magrittr_1.5            R6_2.2.2               
## [69] gplots_3.0.1            Hmisc_4.0-3            
## [71] ADGofTest_0.3           DBI_0.7                
## [73] foreign_0.8-69          survival_2.41-3        
## [75] abind_1.4-5             RCurl_1.95-4.8         
## [77] nnet_7.3-12             tibble_1.3.4           
## [79] pspline_1.0-18          KernSmooth_2.23-15     
## [81] rmarkdown_1.6           locfit_1.5-9.1         
## [83] grid_3.4.1              data.table_1.10.4-2    
## [85] blob_1.1.0              digest_0.6.12          
## [87] xtable_1.8-2            tidyr_0.7.1            
## [89] httpuv_1.3.5            numDeriv_2016.8-1      
## [91] munsell_0.4.3           glmnet_2.0-10
```

## References

Chen, Yunshun, Aaron TL Lun, and Gordon K Smyth. 2014. “Differential Expression Analysis of Complex RNA-Seq Experiments Using edgeR.” *In Statistical Analysis of Next Generation Sequence Data. Somnath Datta and Daniel S Nettleton (Eds), Springer, New York*. https://link.springer.com/chapter/10.1007%2F978-3-319-07212-8\_3.

Love, Michael I, Wolfgang Huber, and Simon Anders. 2014. “Moderated Estimation of Fold Change and Dispersion for RNA-Seq Data with DESeq2.” *Genome Biology* 15: 550. https://genomebiology.biomedcentral.com/articles/10.1186/s13059-014-0550-8.

Robinson, Mark D, and Alicia Oshlack. 2010. “A Scaling Normalization Method for Differential Expression Analysis of RNA-Seq Data.” *Genome Biology* 11: R25. https://genomebiology.biomedcentral.com/articles/10.1186/gb-2010-11-3-r25.

Robinson, Mark D, Davis J McCarthy, and Gordon K Smyth. 2010. “edgeR-a Bioconductor Package for Differential Expression Analysis of Digital Gene Expression Data.” *Bioinformatics* 26: 139–40. https://academic.oup.com/bioinformatics/article-lookup/doi/10.1093/bioinformatics/btp616.
